# Modeling and Predicting Heavy-Duty Vehicle Engine-Out and Tailpipe Nitrogen Oxide (*NO*_*x*_) Emissions Using Deep Learning

**DOI:** 10.3389/fmech.2022.840310

**Published:** 2022

**Authors:** Rinav Pillai, Vassilis Triantopoulos, Albert S. Berahas, Matthew Brusstar, Ruonan Sun, Tim Nevius, André L. Boehman

**Affiliations:** 1 Department of Mechanical Engineering, University of Michigan, Ann Arbor, MI, United States,; 2 Plasma Science and Fusion Center, Massachusetts Institute of Technology, Cambridge, MA, United States,; 3 Department of Industrial and Operations Engineering, University of Michigan, Ann Arbor, MI, United States,; 4 National Vehicle and Fuel Emissions Laboratory, U.S. EPA, Ann Arbor, MI, United States,; 5 Horiba Instruments Inc., Saline, MI, United States

**Keywords:** heavy-duty vehicles, nitrogen oxide emissions, data-driven modelling, deep learning, artificial neural networks, optimization

## Abstract

As emissions regulations for transportation become stricter, it is increasingly important to develop accurate nitrogen oxide (*NO*_*x*_) emissions models for heavy-duty vehicles. However, estimation of transient *NO*_*x*_ emissions using physics-based models is challenging due to its highly dynamic nature, which arises from the complex interactions between power demand, engine operation, and exhaust aftertreatment efficiency. As an alternative to physics-based models, a multi-dimensional data-driven approach is proposed as a framework to estimate *NO*_*x*_ emissions across an extensive set of representative engine and exhaust aftertreatment system operating conditions. This paper employs Deep Neural Networks (DNN) to develop two models, an engine-out *NO*_*x*_ and a tailpipe *NO*_*x*_ model, to predict heavy-duty vehicle *NO*_*x*_ emissions. The DNN models were developed using variables that are available from On-board Diagnostics from two datasets, an engine dynamometer and a chassis dynamometer dataset. Results from trained DNN models using the engine dynamometer dataset showed that the proposed approach can predict *NO*_*x*_ emissions with high accuracy, where *R*^2^ scores are higher than 0.99 for both engine-out and tailpipe *NO*_*x*_ models on cold/hot Federal Test Procedure (FTP) and Ramped Mode Cycle (RMC) data. Similarly, the engine-out and tailpipe *NO*_*x*_ models using the chassis dynamometer dataset achieved *R*^2^ scores of 0.97 and 0.93, respectively. All models developed in this study have a mean absolute error percentage of approximately 1% relative to maximum *NO*_*x*_ in the datasets, which is comparable to that of physical *NO*_*x*_ emissions measurement analyzers. The input feature importance studies conducted in this work indicate that high accuracy DNN models (*R*^2^ = 0.92–0.95) could be developed by utilizing minimal significant engine and aftertreatment inputs. This study also demonstrates that DNN *NO*_*x*_ emissions models can be very effective tools for fault detection in Selective Catalytic Reduction (SCR) systems.

## 1 INTRODUCTION

Heavy-duty vehicles employ compression ignition engines due to their high power density, reliability and powertrain efficiency. Even with the anticipated changes in Greenhouse Gas regulations, diesel engine-powered trucks will continue to be used in heavy-duty transportation for several years, especially in the legacy fleet (EPA, 2021b). Also, the heavy-duty transportation sector is more challenging to electrify due to the need for high energy storage, fast charging rates and high ranges for long-haul movement of goods (Askin et al., 2015; Brown et al., 2020). However, diesel engines emit significant amounts of *NO*_*x*_ (nitric oxide and nitrogen dioxide) which is designated as a criteria pollutant by the EPA (Winkler et al., 2018), and has been shown to cause respiratory illness such as asthma and chronic lung disease upon prolonged exposure. *NO*_*x*_ is also a contributor to the formation of smog, acid rain and ozone at ground levels (Boningari and Smirniotis, 2016). Stringent emissions regulations have therefore been put in place to curb vehicular *NO*_*x*_ emissions (EPA, 2021b). This has put tremendous pressure on the diesel engine industry to design and develop technologies that limit *NO*_*x*_ emissions from the engine and from the tailpipe using exhaust aftertreatment systems. Accurate estimation of instantaneous engine-out *NO*_*x*_ emissions has therefore become essential to improve engine control strategies for *NO*_*x*_ reduction. From a regulations perspective, accurate models for tailpipe *NO*_*x*_ predictions are important in understanding the potential for future emissions reductions, and as a tool for identifying possible modes of non-compliance during in-use operation.

Formation of engine-out *NO*_*x*_ is the result of complex chemical reactions at high temperature within the combustion chamber, and therefore strongly depends on the engine operating condition. On the other hand, tailpipe *NO*_*x*_ emissions are highly dependent on the performance of the Selective Catalytic Reduction (SCR) aftertreatment system. Past studies have made use of thermophysical and chemical models to estimate *NO*_*x*_ emissions. Mentink et al. (2017) developed a virtual engine-out *NO*_*x*_ sensor using a physics-based nitric oxide (*NO*) formation model and an empirical correlation to determine nitrogen dioxide (*NO*_2_) fraction of *NO*_*x*_. A semi-empirical two-zone model was developed by Provataris et al. (2017) that made use of measured in-cylinder pressure data and a physics-based model to estimate *NO* formation in the combustion chamber. Camporeale et al. (2017) used in-cylinder pressure sensor signal to create a grey-box *NO*_*x*_ raw emissions model. The model uses combustion parameters such as adiabatic flame temperature and heat release rate to estimate engine-out *NO*_*x*_ emissions. Multiple studies have also used computational fluid dynamics models to model the changes in temperature and composition in the combustion chamber to better estimate *NO*_*x*_ emissions production (Mobasheri et al., 2012; Dahifale and Patil, 2017). These models use first principles to estimate *NO*_*x*_ emissions and therefore can achieve high extrapolation capabilities. However, they require high computational time and cost, large number of assumptions and the need for laborious manual configuration for different engines.

In the past several years, increasing large quantities of data is being collected through engine and chassis dynamometer laboratory tests due to complex powertrain units with greater number of actuators and finer control. This has led to growing interest in the use of machine learning to develop predictive models for *NO*_*x*_ emissions. Using machine learning, accurate data-driven models can be developed without requiring explicit solution to the governing equations that describe the physics of the system. Selvam et al. (2021) used measurements from On-board Diagnostics (OBD) sensors to calculate combustion variables like adiabatic flame temperature, oxygen concentration and combustion time. These variables were then used as inputs to an ensemble based method called Random Forests to estimate engine-out *NO*_*x*_ emissions for five different heavy-duty engines. The model was evaluated to have an average *R*^2^ value of 0.72 and a mean absolute error (MAE) of 78 parts per million (ppm). A hybrid model consisting of a physics-based model and a machine learning approach was proposed by Mohammad et al. (2021). The model combined a physical and chemical model developed in GT-Suite with a Support Vector Machine and Feed-Forward Artificial Neural Network (FFNN). The model was validated using 772 steady state operating points for a 13L heavy-duty diesel engine and showed good accuracy with an *R*^2^ score of 0.99 and root mean square error (RMSE) of 23 ppm. However, this model was not tested on transient operating conditions. Johri and Filipi (2014) describes the development of a Neuro-Fuzzy Model to predict transient *NO*_*x*_ and soot emissions. This model divides the problem into multiple sub-problems which are individually identified using a simpler class of models. Polynomial and neural network models were used as choices for the local models with validity functions that determine the regions of input space where the local model is active. The model was tested on Unites States Federal city-driving schedule (FTP75) cycle data for a 6.4L heavy-duty engine. The model predictions were in good agreement with the total cumulative *NO*_*x*_ measured over the test cycle.

Deep Learning has been shown to be adept at discovering intricate structures in high-dimensional data and has applications in various domains such as science, business and government (LeCun et al., 2015; Goodfellow et al., 2016). A virtual *NO*_*x*_ sensor using Recurrent Neural Network (RNN) was proposed by Arsie et al. (2013). Data for training was collected by running different test cycles like the New European Driving Cycle (NEDC) on a 1.3L light-duty diesel engine. Engine Control Unit (ECU) variables including engine speed, air mass flow, boost pressure, fuel mass, start of injection (SOI) and air fuel ratio (AFR) were used as inputs to the network. Pruning techniques were used to improve generalization capability of the model. The authors reported *R*^2^ values between 0.83–0.91 for different test sets with RMSE values ranging from 47–122 ppm. Fischer (2013) developed a virtual *NO*_*x*_ sensor for a 2.2L light-duty diesel engine using Self-Organizing Map algorithm—a type of ANN which makes use of a selector and estimator layer. Six input parameters including engine speed, fuel quantity, lambda, air mass flow, boost pressure and exhaust gas temperature were used to estimate engine-out *NO*_*x*_. The model showed good accuracy on the Artemis Urban Test Cycle with an error of 1.57% between the total measured and predicted *NO*_*x*_ over the cycle. Zhang et al. (2015) proposed using an ANN model to predict engine-out *NO*_*x*_ emissions using ECU variables like engine speed, torque, injection timing, air flow rate, rail pressure and oil temperature as inputs. The train and test data consisted of a “Chirp” cycle, Hot and Cold Start drive cycles collected from a 2L light-duty diesel engine. Total *NO*_*x*_ estimated from the model deviated on an average about 5.8% from the measured total *NO*_*x*_ over the test cycle. Bellone et al. (2020) compared a Convolutional Neural Network (CNN) and Long-Short Term Memory (LSTM) network models to predict engine-out *NO*_*x*_ emission, soot and fuel consumption for a heavy-duty 8L diesel engine. Input parameters included ECU variables such as engine speed, fuel flow/cyl., injection angle, rail pressure, wastegate position, exhaust gas recirculation (EGR) position, exhaust temperature, main, post and pre-injection quantity, inlet pressure, pre-injection angle and throttle position. The CNN model captured 98.64% of the total test cycle *NO*_*x*_ emissions with an *R*^2^ of 0.993, while the LSTM model captured 99% of the total test cycle *NO*_*x*_ emissions with and *R*^2^ of 0.995.

Shin et al. (2020) developed an engine-out *NO*_*x*_ emissions model for 1.6L light-duty diesel engine using Deep Neural Networks (DNN) by training the model on the Worldwide Harmonized Light Vehicles Test Procedure (WLTP) cycle. They used Bayesian hyperparameter optimization to find the optimal DNN architecture. The accuracy of the model was indicated by an *R*^2^ value of 0.9675 and MAE of 17 ppm using 14 input variables from the ECU. Yu et al. (2021) presented a method for estimating tailpipe *NO*_*x*_ emissions by complete ensemble empirical model decomposition with adaptive noise and an LSTM network. They used on-road data from the OBD sensors of a diesel bus to train the network. They reported good model accuracy with an *R*^2^ value of 0.98 with RMSE of 46.11 ppm on the test data. A steady state engine-out *NO*_*x*_ emissions model was proposed by Lee et al. (2021) using a DNN model. The model used 8 ECU parameters including engine speed, brake mean effective pressure, EGR rate, air mass, fuel mass, injection timing, boost pressure and injection pressure as inputs. 696 steady state conditions were evaluated using the model with good accuracy indicated by an *R*^2^ of 0.98.

However, the previously conducted studies have developed DNN models for engine-out and tailpipe *NO*_*x*_ emissions without taking into account the effect of SCR performance which is an essential component affecting overall *NO*_*x*_ production. With the light-duty industry expected to be increasingly electrified in the near future, more emphasis also needs to be placed on developing accurate models for estimation of heavy-duty diesel engine *NO*_*x*_ emissions - both engine-out and tailpipe. Additionally, many of the models also make use of ECU variables, such as fuel injection timing, swirl ratio, and injection angle (Bellone et al., 2020; Shin et al., 2020; Lee et al., 2021), which may not be readily available except with proprietary access. Deep Learning models can be highly accurate, but are inherently considered to be black-box models, and therefore it is difficult to interpret their predictions. However, it is crucial to understand for example why *NO*_*x*_ emissions are higher than expected under given engine operating conditions. Multiple papers have used percent of *NO*_*x*_ captured or total test cycle *NO*_*x*_ error as an evaluation metric for *NO*_*x*_ prediction using DNN (Fischer, 2013; Johri and Filipi, 2014; Bellone et al., 2020). The limitations of using this metric in evaluating DNN model accuracy for predicting *NO*_*x*_ emissions has been discussed in the current work and new improved instantaneous error metrics for this application have been proposed.

This work tries to address the aforementioned shortcomings of the existing work in the literature by developing accurate engine-out *NO*_*x*_ and tailpipe *NO*_*x*_ models using DNN. The DNN models were trained and tested using two different datasets - an engine-aftertreatment dynamometer dataset and a chassis dynamometer dataset on two 6.7L heavy-duty bus engines (different model years) using non-proprietary variables that are available from the OBD as model inputs. The outline and contributions of this work are as follows:

- Application of DNN to develop engine-out and tailpipe *NO*_*x*_ emissions models for heavy-duty diesel engines using physics inspired inputs readily available from the OBD, while demonstrating high accuracy on both train and test datasets.
- Development of a DNN model for tailpipe *NO*_*x*_ emissions using SCR aftertreatment information such as SCR inlet and outlet temperatures and exhaust mass flow rate that captures the effect of SCR performance on tailpipe *NO*_*x*_ emissions.
- Analysis and development of holistic error metrics that help visualize instantaneous as well as total *NO*_*x*_ emissions prediction errors of DNN *NO*_*x*_ models over the DNN training process.
- Interpretability study of models to enhance the physical understanding of *NO*_*x*_ emissions estimation using DNN. Evaluation of model accuracy using minimal number of “relatively important” input parameters physically affecting production of *NO*_*x*_ emissions, thereby illustrating DNN model interpretation of complex transient *NO*_*x*_ emissions.
- Detailed analysis of a potential application of developed DNN models to fault detection in SCR aftertreatment systems.

### Organization

The paper is organized as follows. In Section 2 we discuss engine-out and tailpipe *NO*_*x*_ formation, our choice of input features, and the data. Our research methodology is described in Section 3. In Section 4 we present our results followed by an in depth discussion of input feature importance and potential application of developed DNN models in Section 5. We conclude with final remarks in Section 6.

## 2 INPUT FEATURES AND DATA DESCRIPTION

In this section, the thermophysical and chemical phenomena affecting production of engine-out and tailpipe *NO*_*x*_ emissions are explained, which lays the groundwork for the selection of the input parameters for each models. A detailed description of the datasets used to train and test the DNN models is also provided.

### 2.1 Input Feature Selection

In the current study, a DNN model using physics inspired features as inputs has been developed. Therefore, significant engine and vehicle parameters from literature that affect engine-out and tailpipe *NO*_*x*_ formation were measured in the tests conducted to develop the datasets used to train the DNN models, while also taking into consideration their ease of availability from vehicle OBD. A brief description of engine-out *NO*_*x*_ and tailpipe *NO*_*x*_ formation has been provided in the following sections to explain the different inputs selected for each model.

#### 2.1.1 Engine-Out NOx Formation

*NO*_*x*_ is composed of nitric oxide (*NO*) and nitrogen dioxide (*NO*_2_). Diesel engine *NO* formation is described by Three mechanisms - Thermal *NO*, Prompt *NO* and Fuel *NO* (Heywood, 2019). Prompt *NO* is formed in fuel-rich conditions and is not highly temperature dependent. Fuel *NO* formation is dependent on the presence of nitrogen-based compounds in the fuel. However, the primary mechanism for diesel *NO* formation within the combustion chamber is defined by the Extended Zeldovich Mechanism (Lavoie et al., 1970) referred to as Thermal *NO*. Thermal *NO* formation is due to oxidation of nitrogen in the air. The principal reactions governing the formation of Thermal *NO* are given by

(1) $$N_{2}\;+\;O\;=\;NO\;+\;N$$

(2) $$N\;+\;O_{2}\;=\;NO\;+\;O$$

(3) N + OH = NO + H.

These reactions ((1), (2), (3)) are highly dependent on the combustion temperature (> 2000 K), in-cylinder oxygen (*O*_2_) concentrations, and residence time of the reacting mixture at peak temperatures and lean air-fuel mixtures (Bowman, 1975). *NO*_2_ on the other hand is formed due to partial oxidation of *NO* further downstream of the cylinder which can be explained by the following reaction (4) (Merryman and Levy, 1975):

(4) $$NO\;+\;HO_{2}\;=\;NO_{2}\;+\;OH.$$

Engine-out *NO*_*x*_ formation is primarily controlled by the temperature of the burned gas and *O*_2_ concentration in the combustion chamber. These parameters vary based on different engine operating and control variables such as intake air mass flow rate, fuel flow rate, intake manifold temperature and pressure, engine speed and load. Therefore, these variables were selected as inputs for modeling engine-out *NO*_*x*_. Exhaust gas recirculation (EGR) is an important engine-out *NO*_*x*_ control strategy typically employed on diesel engines. The introduction of EGR, which is composed primarily of nitrogen (*N*_2_), carbon dioxide (*CO*_2_), and water (*H*_2_*O*), displaces air in the cylinder, and results in lower *NO*_*x*_ formation. The primary mechanisms for the decrease in *NO*_*x*_ formation due to EGR are the reduction in the mixture’s oxygen concentration, and decrease in the combustion temperatures due to presence of higher specific heat capacity triatomic molecules. As a result, EGR mass flow rate was also included as an input to the DNN when available.

#### 2.1.2 Tailpipe NOx Emissions

Tailpipe *NO*_*x*_ emissions are highly dependent on the performance of the SCR. SCR uses ammonia (*NH*_3_) in the form of aqueous urea as a *NO*_*x*_ reduction reagent to convert *NO*_*x*_ to *N*_2_ and *H*_2_*O*. However, the dominant catalytic reactions require a certain optimal temperature range (520–725 K) (Khair and Majewski, 2006). There are three important SCR reactions given by (Koebel et al., 2002):

(5) $$4NO\;+\;4NH_{3}\;+\;O_{2}\;=\;4N_{2}\;+\;6H_{2}O$$

(6) $$4NH_{3}\;+\;2NO\;+\;2NO_{2}\;=\;4N_{2}+6H_{2}O$$

(7) $$4NH_{3}\;+\;3NO_{2}\;=\;3.5N_{2}+6H_{2}O.$$

The standard SCR reaction (5) occurs in a *NO* dominant environment and requires high temperature. At lower SCR temperatures, a faster reaction rate conversion (6) takes place using equimolar amounts of *NO* and *NO*_2_. This helps to improve SCR performance at lower SCR temperatures. However, an excess of *NO*_2_ results in a slower reaction rate reaction (7). Therefore, SCR conversion efficiency is highly dependent on the ratio of *NO*_2_ to *NO* entering the SCR.

The SCR performance is greatly influenced by the residence time available for reactants in the optimal temperature range, which is a function of the space velocity. The space velocity of the reactor is a function of the measured exhaust gas flow rate through the SCR. The rate of *NO*_*x*_ removal is also dependent on the inlet *NO*_*x*_ concentration, as *NO*_*x*_ levels determine the rate of reactions (Heywood, 2019). Therefore, exhaust aftertreatment variables such as SCR inlet and outlet temperatures, exhaust mass flow rate and measured engine-out *NO*_*x*_ were considered as inputs to the tailpipe *NO*_*x*_ DNN model. Coolant temperature was also included as an input to the model to provide information regarding cold start or hot start conditions for the test cycles. Other variables affecting SCR performance such as urea injection quantity and timing, and ammonia (*NH*_3_) slip were not considered, as they are not readily available from the OBD data without proprietary access.

### 2.2 Data Sources and Dynamometer Test Cycles

Data was collected from two different sources: engine dynamometer testing and chassis dynamometer testing. A 6.7L heavy-duty bus engine (different model years) was tested on an engine and chassis dynamometer using multiple dynamometer test cycles representative of urban, highway, idle, transient and cold start conditions, thereby comprehensively encompassing known sources of transient *NO*_*x*_ emissions.

#### 2.2.1 Engine Dynamometer Data and Test Cycles

**FIGURE 1 |:**
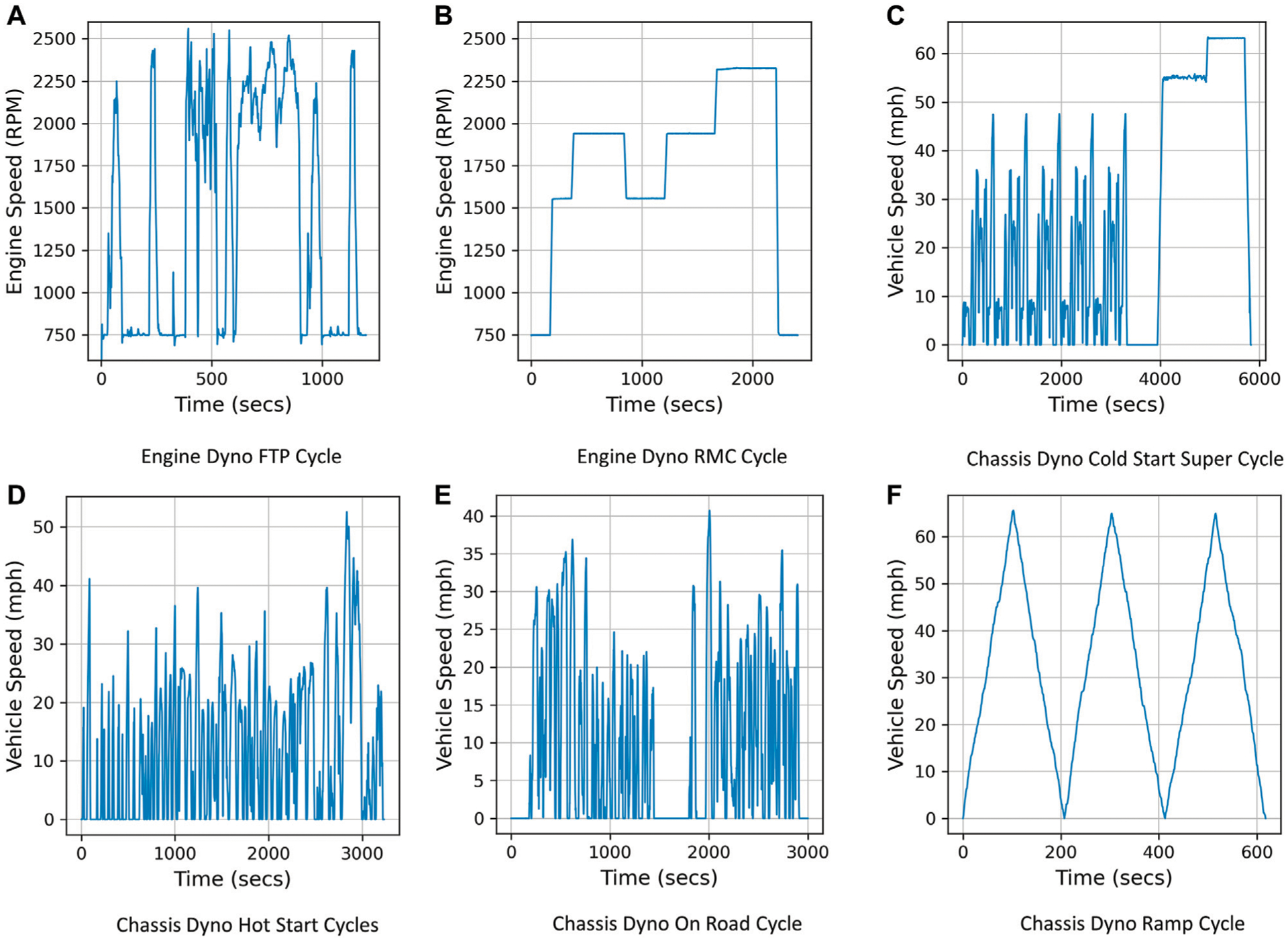
Engine and Chassis dynamometer test cycles used to develop datasets for Deep Learning *NO*_*x*_ Models. **(A)** Engine Dyno FTP Cycle. **(B)** Engine Dyno RMC Cycle. **(C)** Chassis Dyno Cold Start Super Cycle. **(D)** Chassis Dyno Hot Start Cycles. **(E)** Chassis Dyno On Road Cycle. **(F)** Chassis Dyno Ramp Cycle.

Engine dynamometer testing was conducted at the United States EPA, National Vehicle and Fuel Emissions Laboratory (NVFEL), Ann Arbor, on a 6.7L heavy-duty engine (2010) using certification diesel fuel. Test data included engine parameters as well as after-treatment parameters as described in Section 2.1. Both engine-out and tailpipe-out *NO*_*x*_ emissions were measured using exhaust gas analyzers at 10 Hz frequency. All parameters measured using test cell instruments were also recorded at 10 Hz frequency. The dynamometer test cycles included three separate runs of a cold Federal Test Procedure (FTP) cycle, a hot FTP cycle and a Ramped Mode Cycle (RMC) as shown in Figures 1A,B. These tests encompass a variety of engine operating conditions including cold-start, hot-start and transient. This dataset will be referred to as Dataset 1 in this paper. Dataset 1 had a total of 127,223 samples for training the DNN after pre-processing.

In literature, data for testing DNN *NO*_*x*_ models was found to be split from the dataset in two different ways: keeping a separate run of a complete test cycle as test set (Arsie et al., 2013; Fischer, 2013; Zhang et al., 2015; Bellone et al., 2020) or randomly selecting points from the dataset as test set (Shin et al., 2020; Lee et al., 2021; Yu et al., 2021). In this study, both methods were adopted to analyze and compare the effect they had on the model performance. Subsequently. the dataset was first randomly shuffled and then split into train, validation and test sets. 75% of the data was used as train set, of which 25% was used as validation set. The remaining 25% of the total data was used to test the model after training and validation. Splitting of the dataset was also done by using two runs of each test cycle as train set and one run of each test cycle (unseen by the DNN models) as test set.

#### 2.2.2 Chassis Dynamometer Data and Test Cycles

Chassis dynamometer testing was conducted on a hybrid bus that operates on a parallel hybrid architecture using a 6.7L heavy-duty engine (2011) and a 650V nickel-metal hydride battery using certification diesel fuel. The tests were conducted at the Heavy-Duty Chassis Dynamometer Test Facility at the United States EPA NVFEL, Ann Arbor. The facility is capable of simulating on-road conditions for transient and loaded conditions with the help of a road speed modulated vehicle cooling fan with high precision. Continuous tailpipe exhaust measurements were made using a heated dilution tunnel and a Horiba MEXA-One gaseous emissions bench, while engine-out *NO*_*x*_ measurements were sampled directly from the exhaust prior to the SCR, both at 10 Hz frequency. A Cold Start Super Cycle (CSSC), a Hot Start Cycle which was a combination of New York Bus Cycle (NYBC), Orange County Bus Cycle (OCBC), NREL Transient Cycle, an On-Road bus cycle and a Ramp Cycle (Figures 1C–F) were successfully run on the chassis dynamometer to generate a comprehensive dataset encompassing different vehicle operating conditions. OBD data was collected from the bus using Controller Area Network (CAN) at 10 Hz frequency. This included engine and after-treatment parameters similar to the engine dynamometer tests as described in Section 2.1. Battery parameters including state of charge, charging and discharging current were also included as inputs to the engine-out *NO*_*x*_ model to incorporate the influence of hybridization on the *NO*_*x*_ emissions of the bus (Bagheri et al., 2021). This dataset will be referred to as Dataset 2 in this paper.

Dataset 2 had a total of 442,623 samples after pre-processing. As described for Dataset 1, Dataset 2 was also split randomly into equivalent train, validation and test set ratios. The data was also split using 16 runs of complete test cycles as train set and 3 separate runs of test cycles (unseen by the DNN models) as test set. Further details on the train, validation and test splits for both the datasets has been provided in Table 1.

## 3 RESEARCH METHODOLOGY

In this section, the research methodology followed in this paper is described. Specifically, we discuss the model and its associated hyperparameters and the data pre-processing strategy employed.

### 3.1 Feed Forward Deep Neural Network

An Artificial Neural Network (ANN) makes use of representative data to establish empirical relationships between input features and some target output (LeCun et al., 2015; Goodfellow et al., 2016). ANNs have been shown to be adept at establishing complex relationships without the need of strict assumptions or mathematical equations, and as a result have had tremendous success in applications such as image classification (Ciregan et al., 2012), speech recognition (Amodei et al., 2016), and games (Silver et al., 2016); see (Bishop, 1994; Abiodun et al., 2018) for more examples. In its most primitive form, an ANN is a composition of connected neurons arranged in an input layer, possibly a set of hidden layers, and an output layer. Neurons in adjacent layers are connected through edges, or weights. Information flows from the input layer to the output layer through activation functions at each neuron that attempt to capture nonlinearity in the input-output relationship. This process is called “Feed-Forward Propagation”. Training an ANN amounts to adjusting the weights, commonly via the process of “Back Propagation” (Rumelhart et al., 1986), in order to minimize the “objective or loss” function which measures the deviation between the true target output and the predicted output of the network. One Feed-Forward and one Back-Propagation constitute one training “epoch” of the ANN.

Deep Learning Neural Networks (DNN) are a multi-layer manifestation of ANNs (i.e., more than one hidden layer). The predictive power of DNNs is positive correlated with the volume of data and the size of the network, i.e., both the height (number of neurons per layer) and the width (number of layers) (Sun et al., 2017). An important aspect of DNNs is their capability to learn good representations of complex phenomena using “feature learning” (Bengio, 2012). This enables DNNs to learn nonlinear mappings of input features to outputs by generating “high level” features using “low level” (input) training data. These intrinsic characteristics of DNNs make them suitable for modeling and predicting *NO*_*x*_ emissions using simple accessible data.

**FIGURE 2 |:**
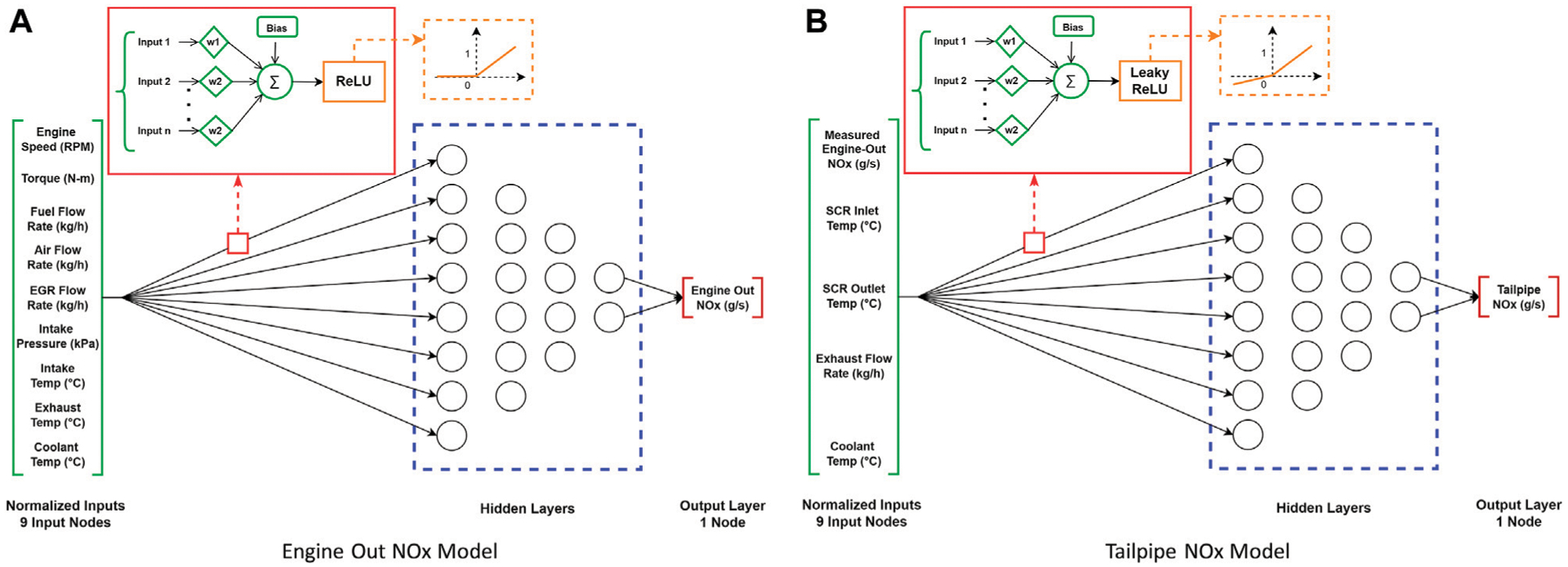
Deep neural network model architecture, inputs/outputs, and activation functions. **(A)** Engine Out NOx Model. **(B)** Tailpipe NOx Model.

In this study, DNNs are used in a supervised learning regression task, i.e., the training data has a labelled output and the output (*NO*_*x*_ emissions) is continuous. The proposed DNNs are trained over multiple epochs to develop *NO*_*x*_ emissions models with high predictive power using the different training datasets (see Section 2.2). The DNNs have multiple “hyperparameters” (e.g., number of hidden layers and nodes, batch size, learning rate) that determine the structure of the neural network and guide the learning process. These parameters are tuned, using exhaustive grid searches (Feurer and Hutter, 2019), to give the best possible performance for a given model, dataset, and computational budget. Prior to training the DNNs, the available datasets are pre-processed in order to help with the training (learning), and as a result increase the predictive power of the DNNs for the given computational budget (Yu et al., 2021); see Section 3.2 for more details. Figure 2 shows the schematic for the DNN engine-out (2a) and tailpipe *NO*_*x*_ (2b) models to describe the DNN architecture.

### 3.2 Data Pre-Processing

Transient data was collected from the engine and chassis dynamometer testing, therefore, it is necessary to eliminate time delay between the input parameters and the measured *NO*_*x*_ emissions before training the DNN models to improve model performance (Arsie et al., 2013; Fischer, 2013; Johri and Filipi, 2014; Zhang et al., 2015). For the engine dynamometer data, the dataset was time-aligned according to 40 Code of Federal Regulations (CFR) Part §1,065 to account for delays in exhaust gas transport and instrument responses (EPA, 2021a). An empirical time constant was derived by cross-correlating the input parameters with the measured *NO*_*x*_ emissions for the chassis dynamometer data for time-alignment of the complete dataset. The effectiveness of the time alignment method was demonstrated with a Pearson’s correlation coefficient of 0.99.

Further, any data points that had negative values due to instrument or calibration errors were removed. This ensures that noisy or unreasonable data does not contaminate the DNN models. The application of DNN to instantaneous *NO*_*x*_ prediction is difficult as it involves the prediction of a continuous variable at every point. To mitigate this issue several studies in the literature have used box plots or median methods to determine and remove “outliers” in the data to improve network performance (Donateo and Filomena, 2020; Yu et al., 2021). In this study, it was found that eliminating outliers also removed a large number of “peak” *NO*_*x*_ conditions which are significant in predicting different transient and “rare” events that occur during vehicle operations. Thus, in order to promote robustness in the DNN models, in this study, data associated with outliers (e.g., “peak” *NO*_*x*_ conditions) was included in the training.

Each dataset was divided into train, validation and test sets as described in Section 2.2. The train set is used to train the models. The validation set provides an unbiased evaluation of the model’s fit on the training samples while tuning the different hyper-parameters of DNN models. The test set is used to evaluate the final model to determine model accuracy and generalization capability.

Feature Scaling is an essential step in data pre-processing for DNN models. The basis for feature scaling is to transform the data such that all the inputs have similar distributions, i.e., a common scale, and equal importance is given to each variable ensuring that no variable influences the model solely due to magnitude (Kotsiantis et al., 2006). Scaling the features helps with the stability, efficiency and robustness of gradient-based optimization algorithms (Wan, 2019). In this study, normalization was used which shifts and rescales the input values to a range between 0 and 1 (also known as min−max scaling) given by:

$$X_{\text{norm}}\;=\;\frac{X\;-\;X_{\text{min}}}{X_{\text{max}}\;-\;X_{\text{min}}}$$

In all the datasets, min −max scaling was fit on the train set and then used to normalize both the validation and test set. This was done to ensure unbiased testing predictions.

### 3.3 Architecture of DNN Models

An Intel^®^ Core^™^ i7-10750H CPU @ 2.60 GHZ (12 cores) with 16 GB RAM and an NVIDIA GeForce RTX 2060 GPU were used for computation. Python 3.60 programming language was used to develop the model with the help of “Keras” deep learning library using TensorFlow (Abadi et al., 2016) as backend. The Python library “scikit-learn” (Pedregosa et al., 2011) was used for pre-processing data, dataset splitting, hyperparameter grid search and model evaluation using different metrics.

#### 3.3.1 Hyperparameter Selection and Optimization

In this study, hyperparameter optimization, a very important ingredient of DNN training (Feurer and Hutter, 2019), is implemented using a grid search where the search space is defined by a grid of hyperparameter values. Every point in the grid which represents a model configuration is then evaluated for performance using appropriate evaluation metrics. Grid search was performed using the GridSearchCV function in the “scikit-learn” library, which allows to perform cross-validation (Refaeilzadeh et al., 2009) in order to understand the generalization capability of each model configuration being tested. The target hyperparameters for optimization included the number of hidden layers, number of nodes in each hidden layer, learning rate and batch size. The ranges of hyperparameters considered for the two datasets are given in Table 2. Table 3 summarizes the final architecture and hyperparameters for each model.

For the hyperparameter optimization, initially a small DNN network composed of two hidden layers with 20 and 10 nodes, respectively, was set up to reduce computational burden. Some hyperparameters were selected based on optimal values in literature that used DNNs for similar supervised learning regression tasks. The Adam optimizer (Kingma and Ba, 2014), a stochastic first-order diagonally scaled method, was used as the optimization algorithm. Associated with the optimization algorithm, the learning rate (a parameter that controls the change in the DNN model weights), as well as the batch size (the amount of data used in the Forward- and Backward-Propagations) were also tuned. With respect to the DNN model, the Rectified Linear Unit (ReLU) activation function (Nair and Hinton, 2010) was considered appropriate for both the hidden layers and the output layer. ReLU is a piece-wise linear function which outputs the input itself if it is positive, but outputs zero if it is negative. In this application all input values are non-negative, thus, ReLU is an appropriate candidate for the hidden layers.

Moreover, the number of hidden layers and nodes in each hidden layer were optimized using a separate grid search using the optimized learning rate and batch size from the first grid search. The different DNN hidden layer configurations for the grid search were developed using a custom function that utilizes three parameters as inputs to create different DNN hidden layer configurations; number of hidden layers, number of neurons in the first hidden layer and number of neurons in the last hidden layer. Based on the number of hidden layers selected, the function individually selects the number of neurons for first and last hidden layers from the ranges provided in Table 2 and linearly decreases the number of neurons in each layer based on the number of hidden layers. For example, if we consider 5 hidden layers, 200 neurons in the first hidden layer and 20 neurons in the last hidden layer, a DNN hidden layer configuration given by (200,155,110,65,20) is created by the function. Each network in both the grid searches was run for 200 epochs with 5 fold cross-validation (Stone, 1978) to determine the optimal batch size, learning rate and hidden layer configuration based on the optimal mean MSE on the cross-validation tests. Further tests were then carried out on the optimal network architecture determined to evaluate the effect of increasing the width of the network (i.e., number of nodes in each hidden layer) and number of epochs on the performance of the model. The number of epochs for training the network was optimized based on the training and validation loss curves to ensure that there was no overfitting of the model on the train set. Number of epochs was used as a termination criteria for the training of all the models. The optimal hyperparameters based on the results from the grid searches have been summarized in Table 3.

##### Learning Rate Decay

Learning rate decay is a mechanism by which the learning rate (employed by the optimization algorithm) is set and adjusted as the optimization progresses in help with learning. Specifically, during the early stages of training large learning rates are employed to allow for large steps, and in order to avoid spurious local minima. In the latter stages of training, a smaller, more refined learning rate is employed in order to obviate the effects of noise, and in order to converge (to a local minimum). Learning rate decay has been shown empirically to improve model optimization and generalization (Smith, 2018; Ge et al., 2019; You et al., 2019). In this study, learning rate decay was used for all the models.

##### Dropout

Overparametrized DNNs, i.e., DNNs with large number of parameter (weights), are prone to overfitting to the training dataset (Sankararaman et al., 2020). Dropout is a regularization technique often employed in DNN training to mitigate this issue (Srivastava et al., 2014). The technique involves randomly “dropping out” nodes along with their connections from the network during training. Temporarily “deactivating” nodes during training reduces the over-adaptation of the network weights to the training data and leads to improvement in network out-of-sample performance and generalization (Baldi and Sadowski, 2013). The fraction of nodes that are deactivated at every iteration of training (“dropout rate”) is often treated as a hyperparameter. In this study a small dropout rate of 0.1 was applied to the hidden layers of all models during training except for the engine-out *NO*_*x*_ model for Dataset 1.

#### 3.3.2 Loss Function and Evaluation Metrics

In this study, the following loss function and evaluation metrics were used.

##### Mean Squared Error (MSE)

Mean Squared Error (MSE) is the default loss function used in many DNNs for regression problems. It is calculated via

$$MSE\;=\;\frac{1}{n}\overset{n}{\underset{i=1}{\sum }}{\left(y_{i}\;-\;{\hat{y}}_{i}\right)}^{2},$$

and is the average of the squared difference between the true output value (y_i_) and the model’s predicted value (${\hat{y}}_{i}$), also referred to as the prediction error.

##### R-Squared (R^2^)

Coefficient of determination or R-Squared (*R*^2^), defined as

$$R^{2}\;=\;1\;-\;\frac{\sum_{i=1}^{n}{\left(y_{i}\;-\;{\hat{y}}_{i}\right)}^{2}}{\sum_{i=1}^{n}{\left(y_{i}\;-\;\bar{y}\right)}^{2}},$$

where $\bar{y}$ is the mean of the true output value, is a statistical measure used to determine the “goodness of fit”, i.e., how well the predicted *NO*_*x*_ values fit with the true *NO*_*x*_ values. Using *R*^2^ as an evaluation metric indicates the model performance across different points in the *NO*_*x*_ distribution. The higher the *R*^2^ value, the better the model prediction across the *NO*_*x*_ distribution.

##### Total NO_x_ Error

Another evaluation metric that has been used in the literature to measure the predictive accuracy of *NO*_*x*_ emissions models is Total *NO*_*x*_ error (%) (Fischer, 2013; Johri and Filipi, 2014; Bellone et al., 2020). Total *NO*_*x*_ error, defined as

$$\text{Total}\;NO_{x}\;\text{Error}\;(\%)\;=\;\frac{\text{Total }NOx_{\text{predicted}}\;-\;\text{Total }NOx_{\text{actual}}}{\text{Total }NOx_{\text{actual}}}*100,$$

is the difference between total true and predicted *NO*_*x*_ emissions in the dataset. Total cumulative *NO*_*x*_ is calculated for train, validation and test datasets for both true and predicted *NO*_*x*_ values. The percent difference between total true and predicted *NO*_*x*_ over each dataset shows if the model has captured various transient *NO*_*x*_ conditions (both high and low) across the dataset effectively and the percentage of total *NO*_*x*_ emissions captured by the model over the dataset.

##### Instantaneous NO_x_ error

Since the DNN models developed in this study are used to predict instantaneous *NO*_*x*_ emissions, we propose a novel instantaneous *NO*_*x*_ error metric that captures the error at every point in the training (validation, testing) set. The absolute prediction error and percent absolute prediction error (%) for every training point is calculated at every epoch of training and is given by

$$\begin{array}{l} \text{Absolute}\;\text{Prediction}\;{\text{Error}}_{i}\;=\;\left|y_{i}\;-\;{\hat{y}}_{i}\right|\\ \text{Absolute}\;\text{Prediction}\;{\text{Error}}_{i}\;(\%)\;=\;\frac{\left|y_{i}\;-\;{\hat{y}}_{i}\right|}{y_{i}}*100. \end{array}$$

The maximum, minimum and mean absolute prediction error and percent error is then calculated and reported for each epoch over the training set. As the DNN learns to predict *NO*_*x*_ emissions at every instant (data point) in the training set, these error metrics should reduce indicating improvement in the model’s instantaneous *NO*_*x*_ prediction capability.

#### 3.3.3 Output Layer Activation Function for Tailpipe NOx Model

Both engine-out and tailpipe *NO*_*x*_ models have 1 node in the output layer as the models are predicting one continuous non-negative output (engine-out or tailpipe *NO*_*x*_ emissions). ReLU was considered as an appropriate candidate for the output layer for both models. However, an interesting phenomenon was observed while examining model predictions for tailpipe *NO*_*x*_. Namely, the model predicted tailpipe *NO*_*x*_ as “0” for many true labels where the *NO*_*x*_ emissions were smaller than 0.0001 g/s (70% of the training set). This resulted in underprediction of total cycle *NO*_*x*_ emissions since many non-zero values were predicted as zero. Extracting the input values to the output layer ReLU activation function showed negative values for smaller true *NO*_*x*_ labels (< 0.000 1 g/s), which by the nature of ReLU are output as “0”, thereby increasing total *NO*_*x*_ prediction error. Thus, other activation functions like LeakyReLU (Maas et al., 2013) and Exponential Linear Unit (ELU) (Clevert et al., 2015) were tested for the output layer. These activation functions are similar to the ReLU activation function with minor differences (e.g., LeakyReLU has a small slope in the negative region; nonzero gradient when the node is not active). It was found that using LeakyReLU improved the tailpipe *NO*_*x*_ model predictions at lower *NO*_*x*_ values at the expense of predicting some negative *NO*_*x*_ values (< 1% of the dataset). The small negative slope helps to smoothen the hard threshold that ReLU has to output “0” value and therefore helps the models predict better at lower *NO*_*x*_ values. LeakyReLU only outputs negative values for very small *NO*_*x*_ true labels < 10^−5^ g/s). These negative values are negligible when compared to the total training set *NO*_*x*_ predicted (< 0.01%), thereby improving overall tailpipe *NO*_*x*_ DNN model performance.

## 4 RESULTS

In this section, the main results for the engine-out *NO*_*x*_ and tailpipe *NO*_*x*_ models are presented. Table 3 summarizes the final architecture and hyperparameters for each model. The results are divided into the following sub-sections: model evaluation metrics (Section 4.1), error metrics (Section 4.2), and instantaneous actual vs predicted *NO*_*x*_ emissions (Section 4.3), on the train, validation and test sets for each model and dataset.

### 4.1 Model Evaluation Metrics Results

The evaluations metrics used to measure the prediction accuracy and overall performance of the models on the train, validation and test sets are reported in Table 4. Cross-validation (5 fold), a popular tool used in machine learning to evaluate a model prediction and generalization capability (Refaeilzadeh et al., 2009), was employed in this study. The results presented in Table 4 are the average MSE, MAE, *R*^2^ and MAE (%) over the maximum *NO*_*x*_ values over the 5 fold cross-validation for each model along with their respective 95% confidence intervals. Overall, the confidence interval values reported in Table 4 indicate that the models were robust to changes in training inputs.

The selection of evaluation metrics was directed at analyzing different aspects of the model’s *NO*_*x*_ prediction capability. First, MSE and MAE were chosen to evaluate if the models were generalizing well to unseen data, i.e., validation and test sets. For all the models, the training and validation MSE and MAE values are very close indicating good model generalization. The test and validation set MSE and MAE values are comparable indicating model robustness to predicting *NO*_*x*_ for conditions unseen by the model when training.

One of the holistic metrics used in the literature to capture *NO*_*x*_, emissions model accuracy is *R*^2^ (Arsie et al., 2013; Bellone et al., 2020; Shin et al., 2020; Yu et al., 2021). As can be seen from Table 4, *R*^2^ values on train, validation and test sets are high for both engine-out and tailpipe models for Dataset 1 and engine-out model for Dataset 2 even with the inclusion of peak *NO*_*x*_, conditions (Yu et al., 2021). Contrary to (Arsie et al., 2013; Zhang et al., 2015; Bellone et al., 2020; Shin et al., 2020) that used ECU variables which are comprehensive but not easily accessible, the models developed in this study achieved comparable high model accuracy while utilizing only simple OBD, parameters as inputs to the models. The aftertreatment system on the bus used to collect data for Dataset 2 has been subject to a product recall due to a manufacturing defect (EPA, 2018). Therefore, the data is not completely representative of a working SCR, system and subsequently the production of tailpipe *NO*_*x*_, emissions. This could explain the relatively lower *R*^2^ values for the tailpipe *NO*_*x*_, model for this dataset. However, the model still captured a significant portion of the transient tailpipe *NO*_*x*_, emissions as indicated by Test *R*^2^ of 0.9275 shown in Table 4.

Linearity of the models was evaluated using mean absolute error (percent) with respect to the maximum *NO*_*x*_ in the dataset. As can be seen from Table 4, the percent MAE with respect maximum *NO*_*x*_ for all the models are well within 1–2%, which is comparable to the *NO*_*x*_ measurement accuracy of *NO*_*x*_ emissions analyzers which have linearity of 1% of full-scale *NO*_*x*_ (Gluck et al., 2003).

**FIGURE 3 |:**
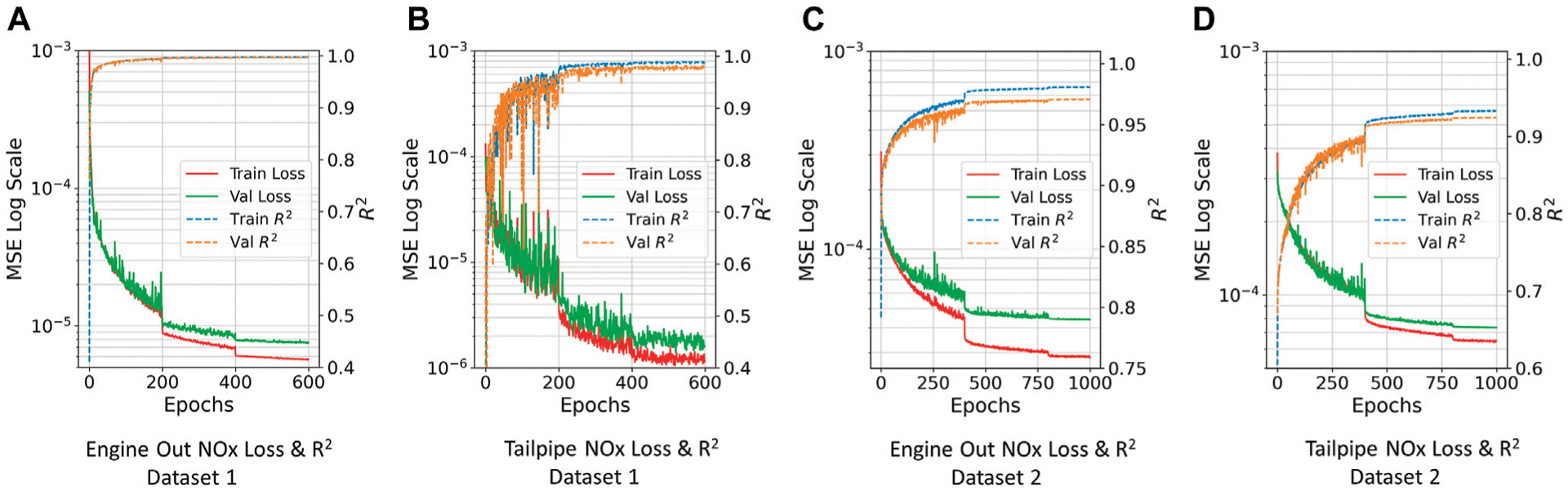
Evolution of MSE and *R*^2^ curves over training and validation data (All Models). **(A)** Engine Out NOx Loss and *R*^2^ Dataset 1. **(B)** Tailpipe NOx Loss and *R*^2^ Dataset 1. **(C)** Engine out NOx Loss & *R*^2^ Dataset 2. **(D)** Tailpipe NOx Loss and *R*^2^ Dataset 2

Figure 3 presents the training and validation MSE (loss) and *R*^2^ curves for each model. These curves help to visualize the progress of the MSE function and *R*^2^ as the network is trained over a set number of epochs. The training loss over the epochs shows how well the model is learning using the given dataset, while the validation loss shows how well the model is generalizing to a smaller validation set that is not being used to train the DNN. For all the models, it can be seen that MSE decreases as the network learns, while *R*^2^ increases which indicates improvement in model prediction. The sudden drops in the loss curves indicate where the learning rate decay was implemented for each model.

**FIGURE 4 |:**
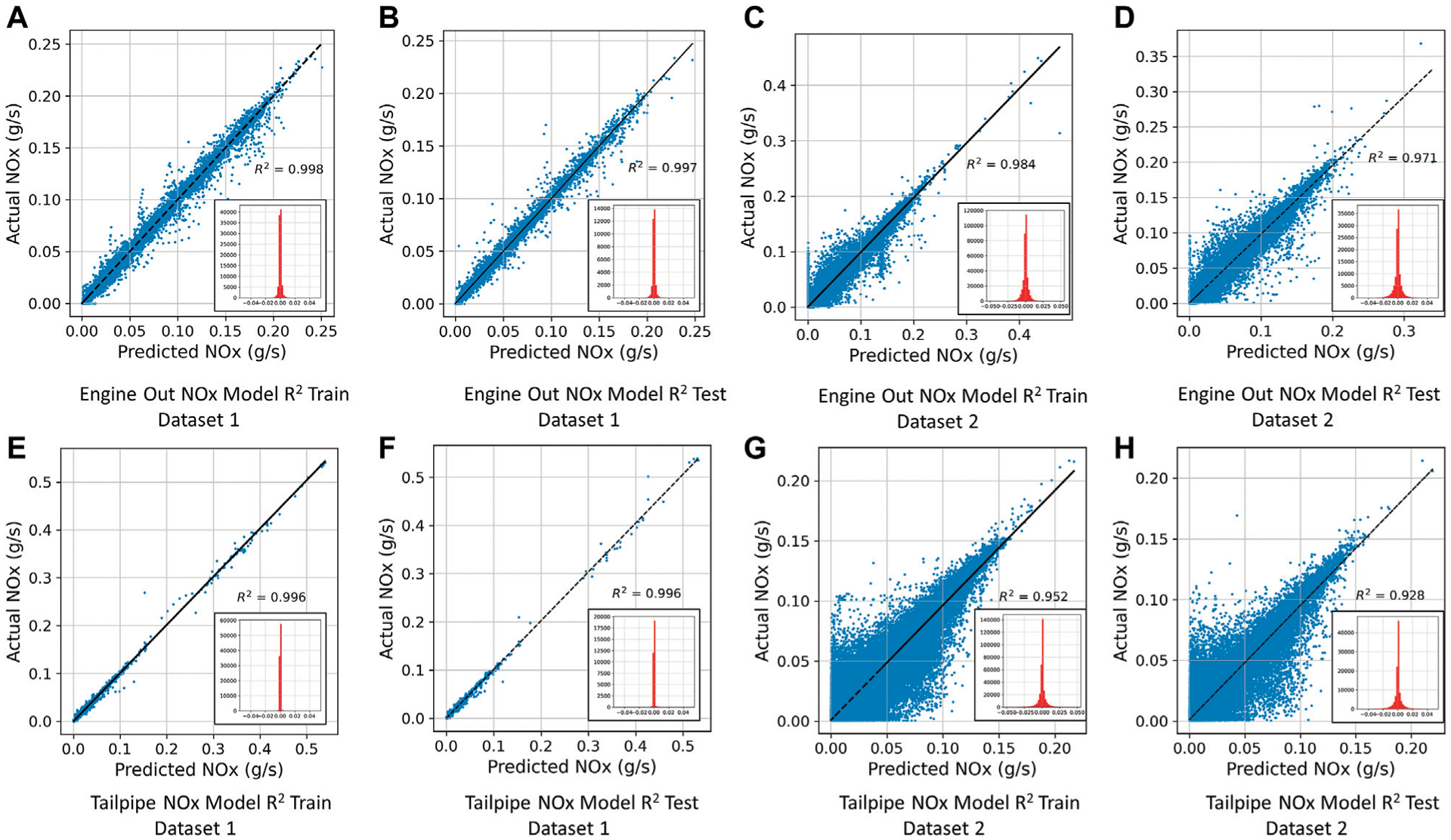
Train and Test *R*^2^ fits with histograms showing distribution of errors (All Models). **(A)**: Engine Out NOx Model *R*^2^ Train Dataset 1. **(B)**: Engine Out NOx Model *R*^2^ Test Dataset 1. **(C)**: Engine Out NOx Model *R*^2^ Train Dataset 2. **(D)**: Engine Out NOx Model *R*^2^ Test Dataset 2. **(E)**: Tailpipe NOx Model *R*^2^ Train Dataset 1. **(F)**: Tailpipe NOx Model *R*^2^ Test Dataset 1. **(G)**: Tailpipe NOx Model *R*^2^ Train Dataset 2. **(H)**: Tailpipe NOx Model *R*^2^ Test Dataset 2.

The results of Regression Analysis (*R*^2^ fit) for train and test datasets of the different models are shown in Figure 4. The models show high degrees of agreement with the measured engine-out and tailpipe *NO*_*x*_ emissions demonstrated by high *R*^2^ values on both train and test sets. The points are well distributed on both sides of the regression fit line which indicates normal distribution of prediction errors with a mean around 0, which is further confirmed by the histogram of errors shown for all the models. The large scatter of points on either side of the regression fit line in Figures 4G,H can be attributed to the large number of data points which causes the “few” outliers in the plot to cover a larger region in the plots.

### 4.2 Error Metrics

**FIGURE 5 |:**
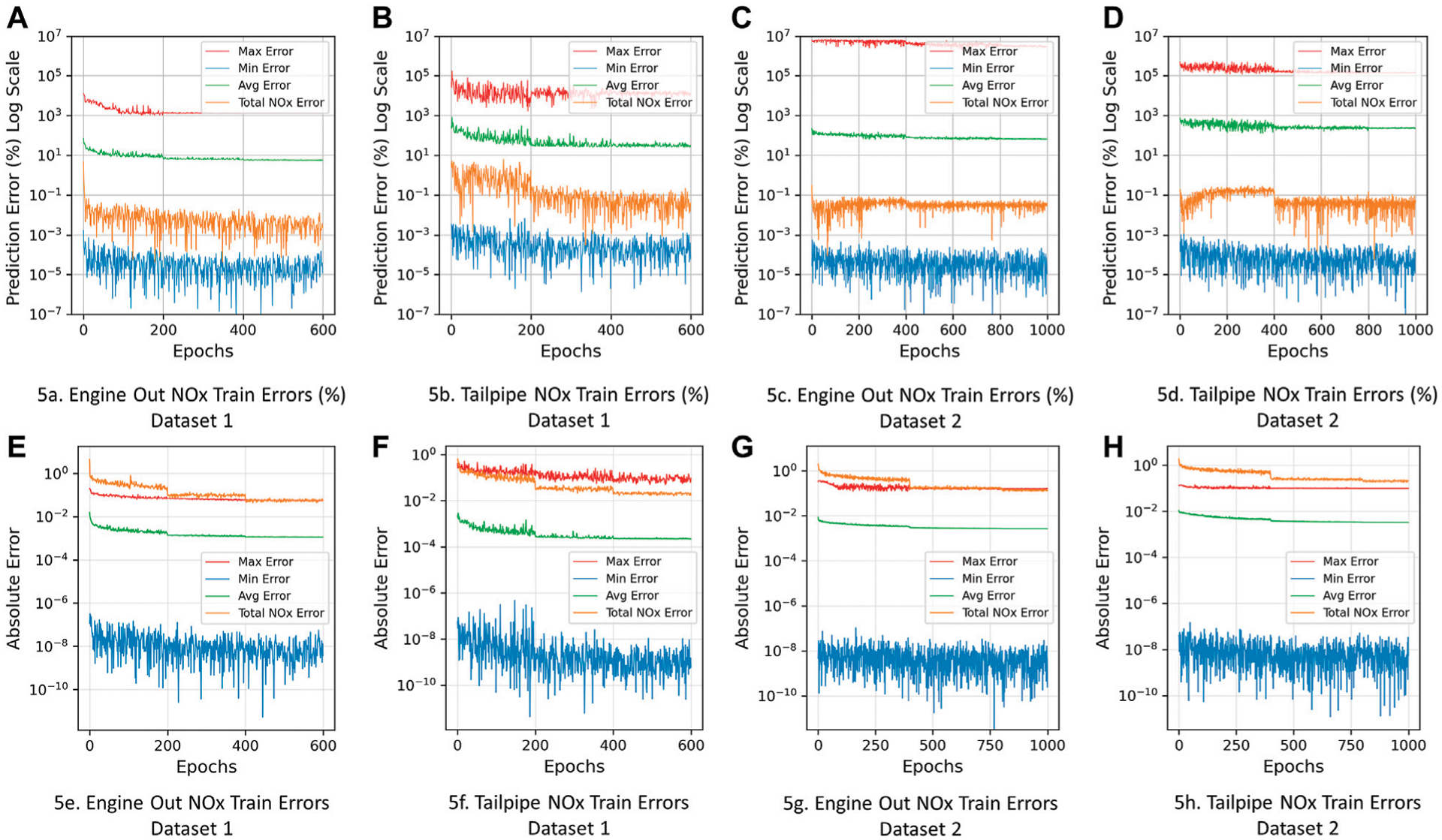
Progression of total *NO*_*x*_ error in comparison with maximum, minimum and mean absolute error over training of all DNN *NO*_*x*_ models. **(A)** Engine Out NOx Train Errors (%) Dataset 1. **(B)** Tailpipe NOx Train Errors (%) Dataset 1. **(C)** Engine Out NOx Train Errors (%) Dataset 2. **(D)** Tailpipe NOx Train Errors (%) Dataset 2. **(E)** Engine Out NOx Train Errors Dataset 1. **(F)** Tailpipe NOx Train Errors Dataset 1. **(G)** Engine Out NOx Train Errors Dataset 2. **(H)** Tailpipe NOx Train Errors Dataset 2.

Figure 5 shows the total *NO*_*x*_ error (%) (Arsie et al., 2013; Johri and Filipi, 2014; Bellone et al., 2020) for train and validation sets (orange line) on a log scale. The error was consistent on average over the training. From Table 4 it can be observed that total *NO*_*x*_ error (%) for lower accuracy models (*R*^2^ = 0.93–0.95) is lower than that of higher accuracy models (*R*^2^ = 0.99). A simple reason for this could be due to the fact that the lower accuracy models overpredict and underpredict instantaneous *NO*_*x*_ more than the higher accuracy models. The results from Table 4 show that there is no clear correlation between *R*^2^ values and the total *NO*_*x*_ error, i.e., higher *R*^2^ models do not show lower total *NO*_*x*_ error. The studies conducted on the models indicate that the magnitude of overprediction and underprediction in lower *R*^2^ models, sufficiently balance out when the cumulative total true and predicted *NO*_*x*_ over the train, validation and test set is calculated, resulting in a lower total *NO*_*x*_ error (see histograms in Figure 4). This suggests that the total *NO*_*x*_ error metric alone is not a good indicator of a model’s instantaneous *NO*_*x*_ predictive capability.

Therefore, in order to better understand the progression of the instantaneous prediction error at every epoch, the (maximum, minimum and mean) absolute prediction error and percent absolute prediction error over the train set were used as additional evaluation merits; see Section 3.3.2 for definition. From Figure 5, it can be seen that all metrics decrease as the model is trained, indicating improvement in model accuracy. The large maximum percent absolute prediction errors (of the order 10^7^) can be attributed to a few very low *NO*_*x*_ emissions points being significantly overpredicted, however, this represents a very small portion of the entire dataset (< 5%). Subsequently, it can be observed that the mean absolute error (%) is low (in the order of 10^1^) indicating higher overall instantaneous prediction capability. Also, it is important to consider the scale of the *NO*_*x*_ emissions while evaluating percentage absolute error. Very small errors in predictions can be blown up when calculating absolute error with respect to the true *NO*_*x*_ emissions which are at the scale of 1E-04 and lower. These absolute errors however are still small when compared to the average value of *NO*_*x*_ emissions in the datasets (0.03–0.05 g/s) as can be seen from Figures 5E–H and Table 4 which shows the mean MAE and percent MAE with respect to the maximum *NO*_*x*_ over the train, validation and test sets.

### 4.3 Actual vs Predicted NOx Emissions

**FIGURE 6 |:**
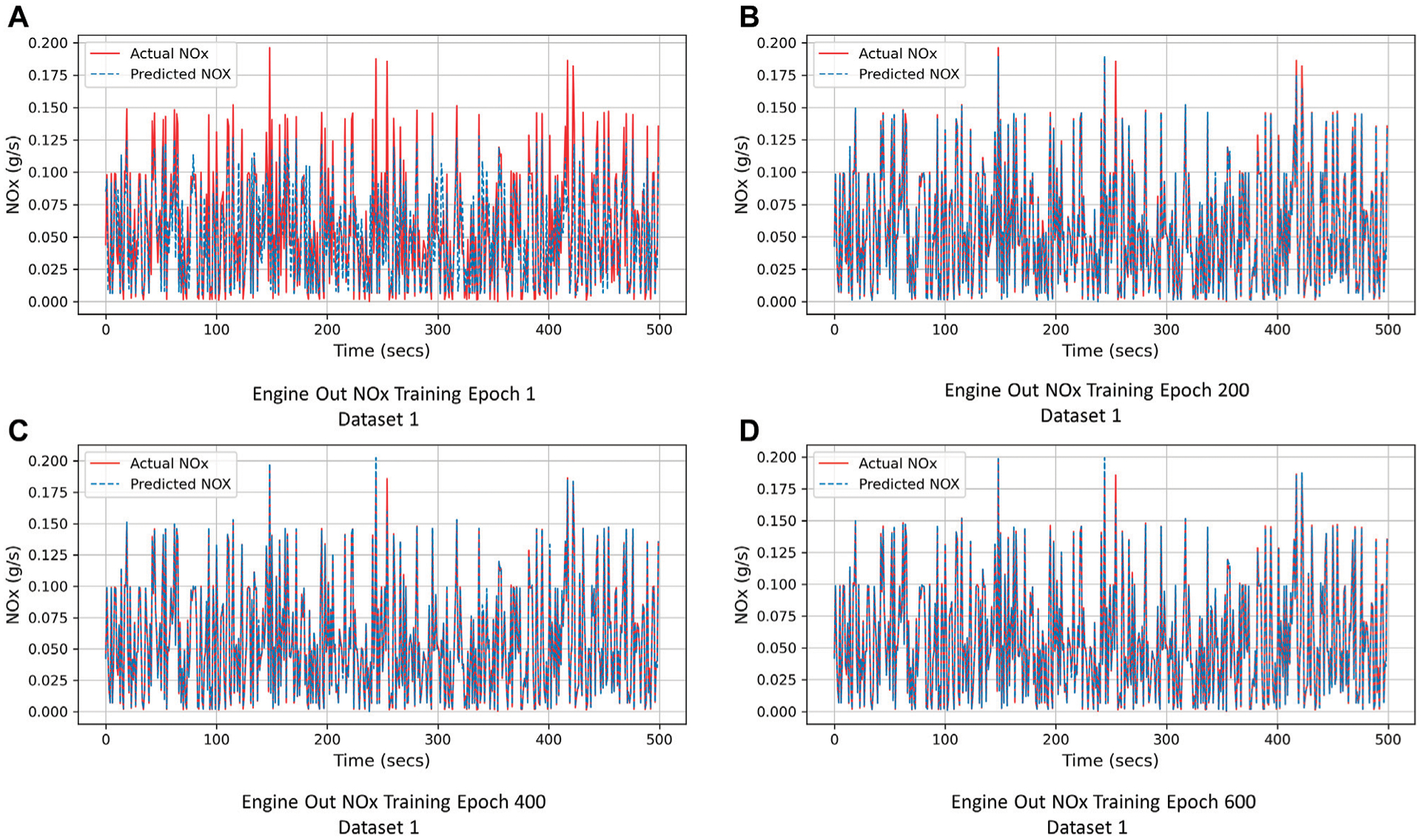
Improvement of instantaneous *NO*_*x*_ prediction over DNN training (Engine-Out *NO*_*x*_ model Dataset 1). **(A)** Engine Out NOx Training Epoch 1 Dataset 1. **(B)** Engine Out NOx Training Epoch 200 Dataset 1. **(C)** Engine Out NOx Training Epoch 400 Dataset 1. **(D)** Engine Out NOx Training Epoch 600 Dataset 1.

**FIGURE 7 |:**
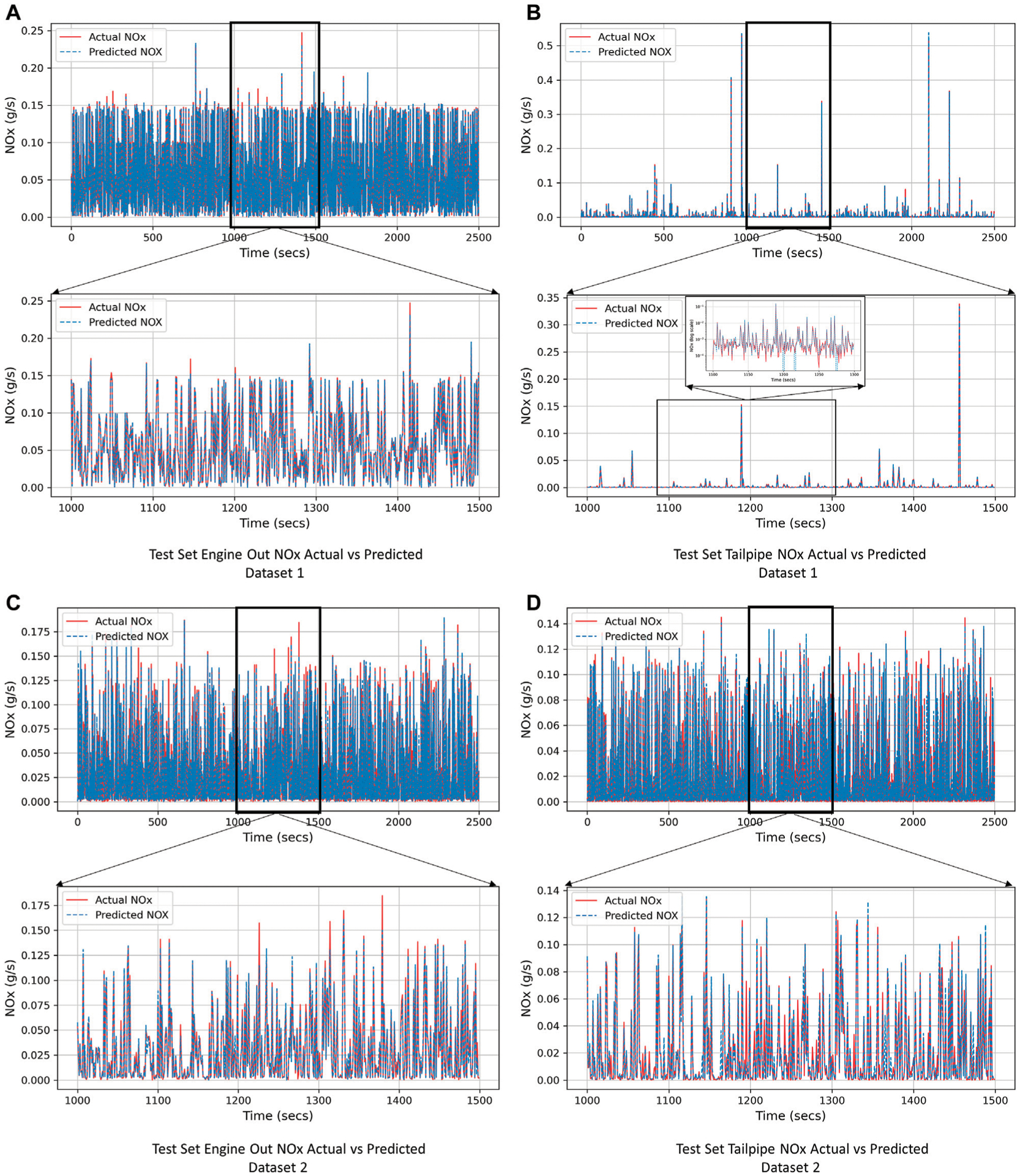
Actual vs Predicted *NO*_*x*_ emissions for a portion of the test set (All Models). **(A)** Test Set Engine Out NOx Actual vs Predicted Dataset 1. **(B)** Test Set Tailpipe NOx Actual vs Predicted Dataset 1. **(C)** Test Set Engine Out NOx Actual vs Predicted Dataset 2. **(D)** Test Set Tailpipe NOx Actual vs Predicted Dataset 2.

Figures 6, 7 depict the actual *NO*_*x*_ emissions in red and the predicted *NO*_*x*_ emissions in blue. Such visualizations aid in understanding the predictive capabilities of the models over the course of training, and clearly highlight points for which the model is overpredicting or underpredicting *NO*_*x*_ emissions.

As an example, Figure 6 depicts the improvement in the predictions of the engine-out *NO*_*x*_ DNN model for Dataset 1, over a small section of the train set as the network learns. Figures 6A–D show the actual and predicted *NO*_*x*_ emissions for the DNN model at epochs 1, 200, 400 and 600 respectively. It can be observed that as the network is trained, subsequently the predictions (dashed blue line) approach the true values of *NO*_*x*_ emissions (red line). The increasing overlap of the two lines in Figures 6B–D indicates significant improvement in the model predictive capabilities over the course of training.

Figure 7 shows the *NO*_*x*_ prediction results for a portion of the test set for all the models. Sections of the test set have been enlarged below each sub-plot to provide more clarity to the profile for measured and predicted *NO*_*x*_ emissions. Figure 7B also includes an enlarged log-scale plot to show the peak tailpipe *NO*_*x*_ predictions more clearly. The DNN models are able to capture both dips and peaks in the engine-out and tailpipe *NO*_*x*_ emissions effectively as observed from the enlarged plots. The DNN models were successfully able to capture high frequency oscillations in previously unseen test data, while having MAE within 1–2% of the full scale of the *NO*_*x*_ emissions available in the dataset. Overall, the results are very promising, and the models appear to be suitable for transient *NO*_*x*_ emissions estimation in engine-out *NO*_*x*_ control and tailpipe *NO*_*x*_ compliance applications.

## 5 DISCUSSION

In this section, three different studies conducted have been described which highlight some important aspects of the application of DNNs to *NO*_*x*_ emissions predictions. First, an analysis of the effect of type of input data split on the accuracy of developed DNN models is presented. Then, insights from an input feature importance study are discussed. Finally, the effectiveness of the developed DNN models to fault-detection in SCR aftertreatment systems is also presented.

### 5.1 Effect of Training Data on Model Accuracy

**FIGURE 8 |:**
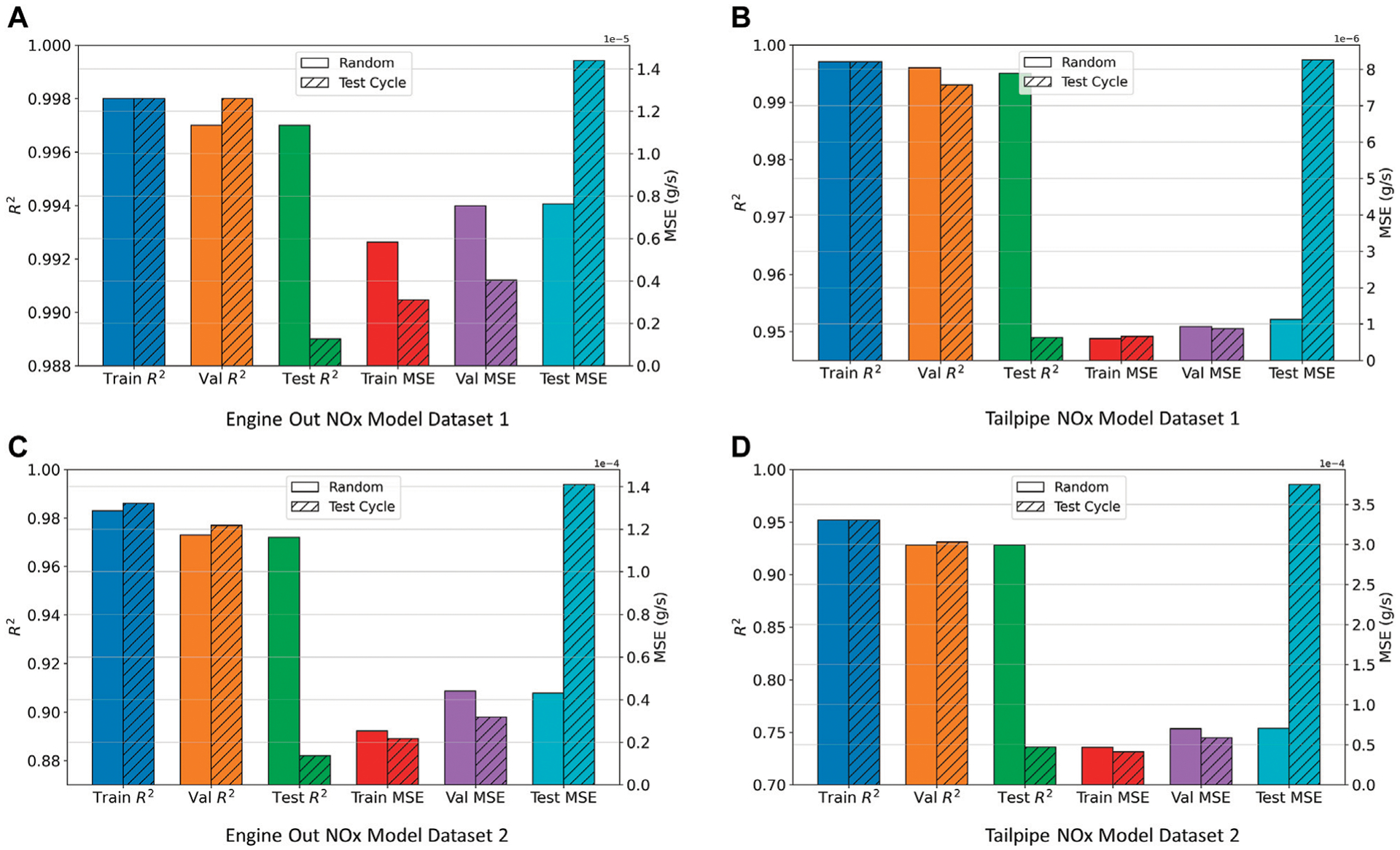
Effect of type of data split on model performance. **(A)** Engine Out NOx Model Dataset 1. **(B)** Tailpipe NOx Model Dataset 1. **(C)** Engine Out NOx Model Dataset 2. **(D)** Tailpipe NOx Model Dataset 2.

In this section, the effect of model training data for *NO*_*x*_ prediction on the DNN model accuracy has been analyzed. DNN models were trained using data split by two different approaches as described in Section 2.2. The results for this experiment on each of the models is presented in Figure 8. It was observed that when the models were trained using randomly selected training data, they had good accuracy (both *R*^2^ and MSE) on train, validation and test sets. However, when the models were trained using complete test cycles and then tested on different (unseen) complete runs of the same test cycles, the models had less prediction accuracy on the test set. However, the models are not overfitting on the training data, as *R*^2^ and MSE of the “hold-out” validation set is comparable to that of the train set, as can be seen from the striped bars in Figure 8.

One explanation for this phenomenon could be that there are variations in *NO*_*x*_ emissions measurements across multiple runs of the same test cycle, either due to instrument measurement deviations or accuracy limits or due to the effect of an engine or aftertreatment parameter that is not included in the input features for the DNN models - for eg. fuel injection pressure and timing, urea injection or *NH*_3_ slip. The results from this study suggest that DNN models trained using randomly selected data, are capable of learning the different variations in *NO*_*x*_ measurements that occur at similar inputs much better than when the model is trained using whole test cycles. The variation in *NO*_*x*_ emissions at the same point in the dataset between the test cycle runs in the train set and the test set are unknown to the models when the data is not split randomly. This could be causing the higher error between the model prediction and the true *NO*_*x*_ measurement in the test cycle. Therefore, to improve model prediction when using multiple runs of whole test cycles to train the DNN, it could be advisable to add more input features to explain the variation in true *NO*_*x*_ measurements. Fuel injection strategies affect the formation of *NO*_*x*_ emissions (Agarwal et al., 2013). SCR performance is also affected by *NH*_3_/*NO*_*x*_ ratio, while NH3 slip could also affect the effectiveness of engine-out to tailpipe *NO*_*x*_ conversion (Girard et al., 2007). Therefore, proprietary ECU parameters such as fuel injection pressure and timing, urea injection timing and quantity and NH_3_ slip could be included as inputs to the DNN models to capture the variation in *NO*_*x*_ emissions.

### 5.2 Input Feature Importance Study

DNN’s are inherently black-box models due to their multi-layer nonlinear architecture. DNN’s are capable of modelling complex problems with high accuracy but at the expense of losing “explainability”, i.e., how transparent the model’s predictions are to a human (Fan et al., 2021). In the application of predicting *NO*_*x*_ emissions, especially for engine control and compliance purposes it is important to discern where and why the model is predicting incorrectly. Therefore, an attempt has been made to determine “important” inputs to the DNN models presented in this paper that affect model prediction. This study does not completely make the DNN models transparent, but tries to gain some understanding into the inner workings of the DNN’s characterization of various engine and aftertreatment parameters to the production of *NO*_*x*_ emissions.

**FIGURE 9 |:**
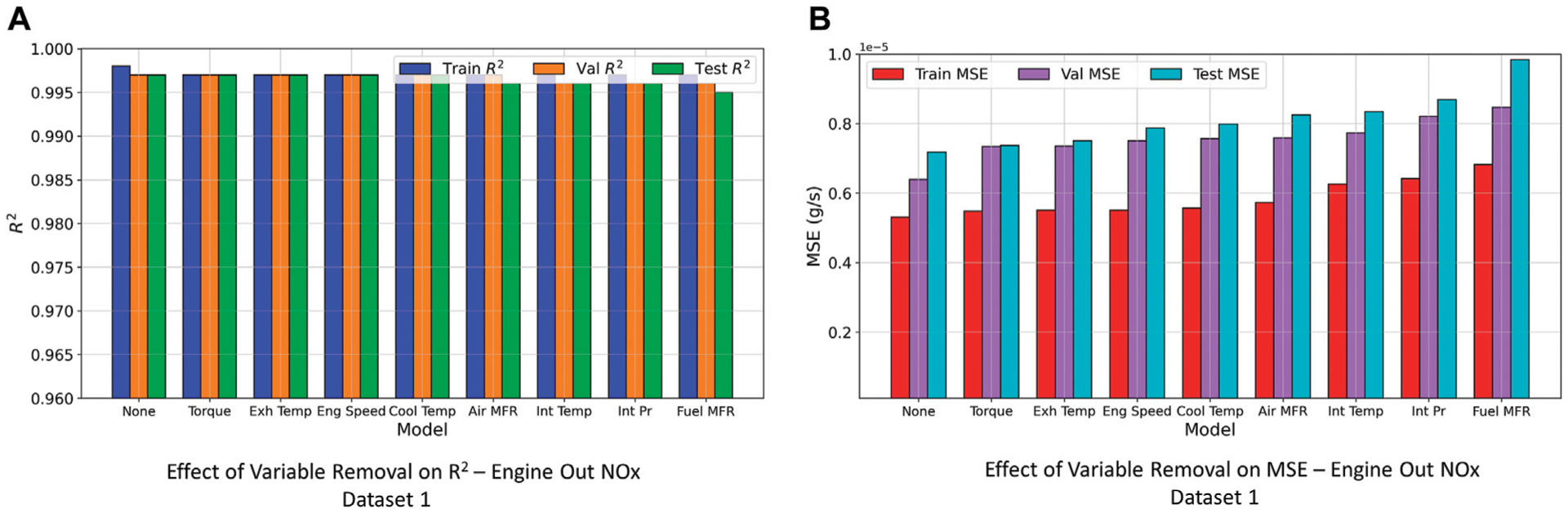
Effect of variable removal on model performance (Engine out *NO*_*x*_ model Dataset 1). **(A)** Effect of Variable Removal on *R*^2^—Engine Out NOx Dataset 1. **(B)** Effect of Variable Removal on MSE—Engine Out NOx Dataset 1.

The DNN models make use of input engine or aftertreatment parameters to learn the complex transient nature of *NO*_*x*_ emissions. Therefore, by removing an input feature that is important to the DNN to learn, would result in decrease in the model accuracy. This was the underlying principle used to develop an understanding of relative importance of engine or aftertreatment variables for the DNN models to predict *NO*_*x*_ emissions. In this study, few (5–9) input features were used to train the models, and as a consequence this method was easier to implement. After the DNN models were trained, the models (with the same hyperparameters and architecture) were trained again by removing one input feature at a time to find the input feature which when removed reduced the model prediction accuracy, i.e., *R*^2^ and MSE. As an example, results for the experiments have been presented for the engine-out *NO*_*x*_ model using Dataset 1 in Figure 9.

However, as can be seen from the *R*^2^ and MSE values for both train and test sets, removal of any single input variable did not seem to affect the model prediction accuracy. The actual physics of *NO*_*x*_ formation and engine operation could provide an explanation for this phenomenon. *NO*_*x*_ emissions are formed due to highly complex chemical and physical phenomena which are affected by parameters that are highly dependent on each other. Therefore, even if one variable or input is removed from the DNN model, information provided by other engine or aftertreatment variables help the DNN to capture the complex process of *NO*_*x*_ formation in diesel engines. The observed independence could also be because the DNN was sufficiently over-parameterized, i.e., number of parameters in the network exceeds the training points (~ 10^6^ model parameters vs ~ 10^5^ training points) Therefore, the network is able to learn from existing input features even if one input feature is removed. The two hypotheses could be responsible in conjunction for the observed independence of model accuracy on the removal of single input features.

Further testing was therefore conducted by removing multiple sets of inputs till a significant reduction in model prediction accuracy was achieved to test the model’s capability to model *NO*_*x*_ emissions with only the most “important” variables. For the engine-out *NO*_*x*_ model, it was observed that just providing engine speed and torque as inputs to the DNN still resulted in model accuracy (*R*^2^) of 0.93. This is consistent with the diesel engine operation—as the engine speed and torque essentially determine the engine operating conditions which is highly related to the production of engine-out *NO*_*x*_ emissions. However, the significant reduction in accuracy suggests that the other input features also contribute to the DNN prediction accuracy on a smaller scale when compared to engine speed and torque. Similarly, using chassis dynamometer data which was collected from a hybrid bus, model accuracy, i.e., *R*^2^ of 0.93 was achieved using engine speed and torque along with state of charge and charging current as input features.

**FIGURE 10 |:**
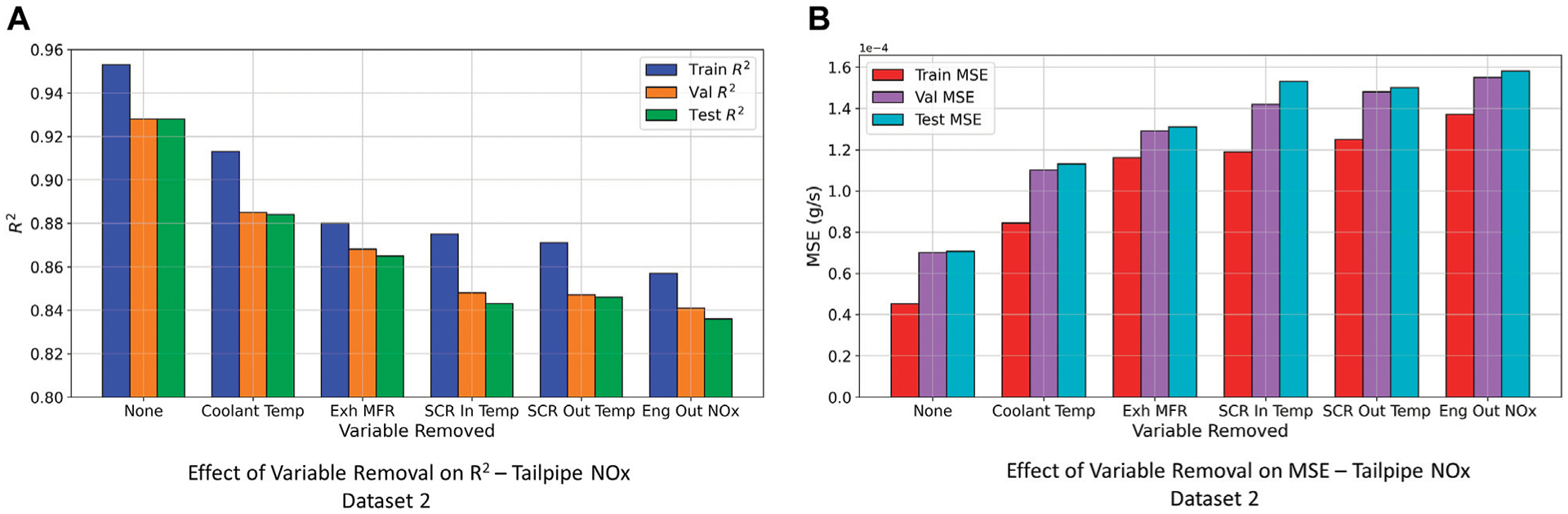
Effect of variable removal on model performance (tailpipe *NO*_*x*_ model Dataset 2). **(A)** Effect of Variable Removal on *R*^2^—Tailpipe NOx Dataset 2. **(B)** Effect of Variable Removal on MSE—Tailpipe NOx Dataset 2.

For the tailpipe *NO*_*x*_ model using the engine dynamometer dataset, utilizing SCR inlet temperature, exhaust mass flow rate and engine-out *NO*_*x*_ as inputs resulted in a model prediction accuracy with *R*^2^ of 0.95 on the test set. However, for the tailpipe *NO*_*x*_ model for the hybrid bus, removing even a single input feature resulted in significant reduction in model prediction accuracy as shown in Figure 10. This could be attributed to incorrect functioning of the SCR system for this hybrid bus (EPA, 2018). Subsequently, the data collected to train this tailpipe *NO*_*x*_ DNN model, is not a true representation of the effect of SCR parameters on tailpipe *NO*_*x*_ emissions. Considering that this particular DNN model is much “deeper” and “wider” than the other tailpipe *NO*_*x*_ DNN model, over-parameterization of the network was inadequate for the network to completely capture incorrect operation of the SCR system.

The variable importance study however suggests that the DNN models are capable of capturing some aspects of the physics of engine-out and tailpipe *NO*_*x*_ emissions without the need for any physical or chemical equations. Important engine and aftertreatment variables guide the DNN models to predict with higher accuracy. The study conducted also demonstrates that utilizing minimal information, i.e., two to four physics inspired inputs, the DNN models developed in this study are capable of capturing complex trends of *NO*_*x*_ emissions in heavy-duty vehicles as indicated by the *R*^2^ values of 0.92 for engine-out and 0.95 for tailpipe *NO*_*x*_ model.

### 5.3 DNN as a Fault Detection Tool for Engine and Aftertreatment System

This section presents an example of the application of DNN models such as the ones developed in this paper for detection of anomalies or faults in diesel vehicles. If DNN models using physics inspired inputs are trained using data from functioning engine and aftertreatment systems, the predictions of the model can compared with data that is obtained from in-use vehicles. This can be applied to detect abnormal *NO*_*x*_ emissions occurring either due to a faulty engine operation, incorrectly operating aftertreatment systems or defeat devices. Fault detection can therefore be performed on an engine-level, as well as, aftertreatment-level.

**FIGURE 11 |:**
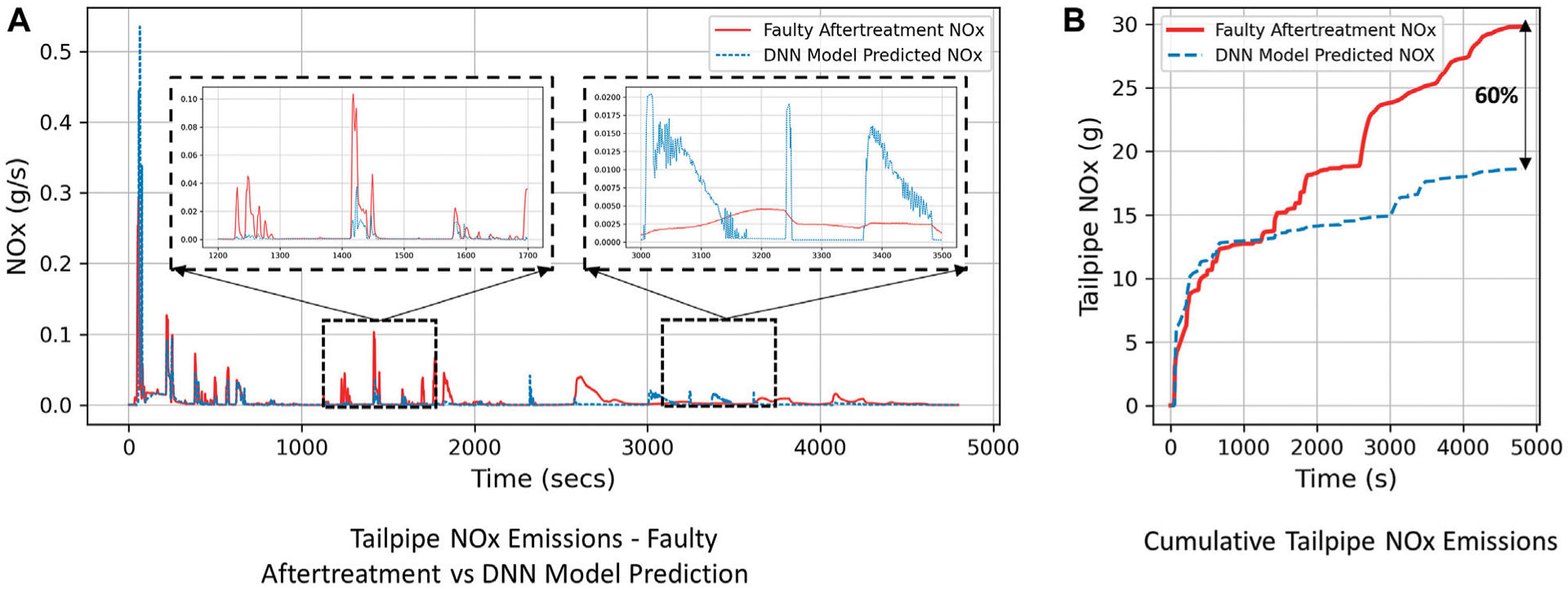
Application of DNN for fault detection in SCR aftertreatment Systems. **(A)** Tailpipe NOx Emission-faulty after treatment vs DNN model prediction. **(B)** Cumulative Tailpipe NOx Emission.

As an example, data was collected from an poorly-functioning aftertreatment (SCR) system for the same engine as the one used for engine dynamometer testing in this paper. The DNN model trained using data from a functioning aftertreatment system was used to predict the tailpipe *NO*_*x*_ emissions for this engine. The tailpipe *NO*_*x*_ emissions predictions for all three engine dynamometer test cycles (Section 2.2) were then compared with the “faulty” aftertreatment system data. In Figure 11A, the blue dashed line indicates the DNN prediction (using functioning aftertreatment system data) and the red line indicates *NO*_*x*_ emissions measurements collected from the faulty aftertreatment system. The cumulative total tailpipe *NO*_*x*_ emissions over the 3 test cycles for the faulty aftertreatment system was 29.81 g while, the DNN model predicted the expected total tailpipe *NO*_*x*_ emissions (if the aftertreatment was functioning correctly) of 18.6 g, as shown in Figure 11B. The engine with a faulty aftertreatment system produced 60% higher tailpipe *NO*_*x*_ emissions which was successfully detected by the DNN model. This example demonstrates the capability of optimized DNN models to detect *NO*_*x*_ emissions anomalies or faults in diesel vehicles and their application for testing and compliance purposes.

### 5.4 Application of DNN Models to Other Heavy-Duty Engines and Fuels

This section discusses the application of similar DNN models to other heavy-duty engines and fuels. Even though the models developed in this paper have been trained on data from one particular engine, it is expected that due to the use of physics inspired features, the models are capable of capturing the significant features and trends that affect transient *NO*_*x*_ emissions in other heavy duty diesel engines. However, from an instantaneous engine-out *NO*_*x*_ perspective, since it is a continuous variable and highly dependent on engine design and calibration and fuel injection strategies, trained model accuracy would be reduced when tested directly on other engines not used to train the models developed in this study. Also, from a tailpipe *NO*_*x*_ model perspective, SCR aftertreatment systems with different catalysts have different *NO*_*x*_ conversion dependencies on the inputs used in the DNN models developed in this study. Therefore, using comprehensive datasets such as the ones developed in this study for other engines and aftertreatment systems, and subsequently applying a similar training process as described in this study should theoretically result in accurate DNN *NO*_*x*_ emissions models for different heavy-duty engines. The current DNN models could also be modified to include other inputs that capture the effect of different engine designs and calibration and SCR catalysts along with comprehensive datasets for other engines to evaluate model performance on other heavy-duty engines.

The type of fuel tested to develop the datasets used to train the DNN models in this study could influence the performance of the trained models. The models in this study were trained using data from engine and chassis dynamometer testing running on certification diesel fuel. Therefore, the dataset used to train the DNN models captures the *NO*_*x*_ emissions trends for certification diesel fuel. Capturing *NO*_*x*_ emissions trends for other fuels such as biodiesel or other higher oxygenated renewable fuels would require subsequent training and optimization using vehicle testing data using these fuels. DNN models developed in this study could be utilized as a base model for initial training using data from a different fuel and then optimized for *NO*_*x*_ emissions prediction. A larger dataset that encompasses *NO*_*x*_ emissions trends using different fuels on a single engine can be used to train similar DNN models with fuel type as an input. The model performance could then be evaluated to capture the influence of fuels on the DNN *NO*_*x*_ emissions predictions.

## 6 CONCLUSIONS AND FUTURE WORK

Deep Neural Network (DNN) models were developed using physics inspired inputs to predict transient heavy-duty diesel engine-out and tailpipe *NO*_*x*_ emissions using engine and aftertreatment variables. The study employed popular and well-established techniques in machine/deep learning to develop engine-out and tailpipe *NO*_*x*_ emissions models with high predictive power. Based on an in-depth analysis of the DNN models for predicting *NO*_*x*_ emissions developed in this study, the following conclusions can be drawn:

1. DNN models using physics inspired inputs are capable of effectively characterizing the complex, nonlinear nature of transient engine-out and tailpipe *NO*_*x*_ emissions. In this study, simple and easily accessible OBD parameters (inputs) were used to develop accurate DNN models. All the models developed in this study have a mean absolute error percentage within 1–2% of the maximum *NO*_*x*_ measurement, which is comparable to physical *NO*_*x*_ emissions measurement analyzer accuracy of 1% of full scale.
2. Novel tailpipe *NO*_*x*_ models developed using SCR aftertreament variables, such as SCR inlet and outlet temperature, engine-out *NO*_*x*_ and exhaust mass flow rate, showed good prediction accuracy (*R*^2^ = 0.99). However, the DNN tailpipe *NO*_*x*_ model developed using data from a faulty SCR system exhibited lower prediction accuracy (*R*^2^ = 0.92) on the test set.
3. This study analyzed the effect of type of dataset splitting on the model accuracy. It was shown that randomly splitting the dataset into train and test sets provides a better understanding of cycle-to-cycle *NO*_*x*_ emissions variation to the DNN model while training—thereby improving model accuracy on the test set. If the DNN models are trained using multiple runs of test cycles as train data, it would be advisable to include more input features that provide additional information to the DNN about the cause of disparity in *NO*_*x*_ emissions for similar test cycles.
4. The feature importance study conducted on the DNN models showed the robustness of the models to removal of single input features while training the network. It was also observed that the DNN models had close understanding of the complex transient nature of *NO*_*x*_ emissions as they were trained using physics inspired input features. Engine-out *NO*_*x*_ models showed good correlation with engine operating conditions like engine speed and torque (*R*^2^ = 0.93), while tailpipe *NO*_*x*_ models exhibited good accuracy even with just three aftertreatment variables as inputs (*R*^2^ = 0.95). Interestingly, the DNN models did not perform equally well when trained using data from a poorly functioning aftertreatment system (*R*^2^ = 0.92). This indicated that when DNN models for *NO*_*x*_ emissions are trained using physics inspired inputs, training data that is not representative of the physics of *NO*_*x*_ emissions formation can lead to relatively poor DNN model performance.
5. This work demonstrated that DNN *NO*_*x*_ emissions models can be very effective tools for fault detection in Selective Catalytic Reduction (SCR) systems. Cumulative *NO*_*x*_ predictions from the DNN model detected that the engine with a faulty aftertreatment (SCR) system produced 60% more total cycle *NO*_*x*_ (g) than the expected *NO*_*x*_ emissions from a functioning aftertreatment (SCR) system.

Future work in this domain will involve the application of similar DNN models to on-road testing data from OBD information and a Portable Emissions Measurement System (PEMS). On-road emissions prediction presents an interesting challenge as environmental variables such as road grade, ambient temperature, pressure and humidity also affect *NO*_*x*_ emissions, but their effects are not necessarily captured in the controlled environment of laboratory testing. Use of DNN models that are trained using physics inspired inputs along with real-world driving effects would help develop models for on-road *NO*_*x*_ prediction, which should reduce the disparities in *NO*_*x*_ emissions between on-road and laboratory type tests. However, measurements from low cost production on-road sensors are less repeatable than those taken from expensive instruments used in engine and chassis dynamometer test cells, and hence it would be challenging to achieve equally high accuracy models using on-road data. The influence of different type of fuels on DNN model *NO*_*x*_ prediction could also be explored by training the models on comprehensive datasets including data from different fuels tested on a heavy-duty vehicle. More heavy-duty engines and aftertreatment systems could be incorporated into the DNN models to assess the model’s robustness in predicting *NO*_*x*_ emissions from different engine sizes and SCR systems. Successful implementation using comprehensive datasets available from chassis and engine dynamometer testing regularly conducted for compliance purposes could result in a database created to measure cumulative *NO*_*x*_ emissions over test cycles for different heavy-duty engines using the DNN models. This would be important to inform the development of future *NO*_*x*_ emissions regulations and for validating real-world emissions measurements against expected performance.

## Figures and Tables

**TABLE 1 |:** Description of datasets (engine and chassis dynamometer. Types of data splits (random and test cycles) and train/validation/test splits.

Dataset	Engine dynamometer		Chassis dynamometer	
Type of Data split	Random	Test cycles	Random	Test cycles
Total Samples	127,223	127,223	442,623	442,623
Train	76,334	64,883	265,574	301,569
Validation	19,083	16,221	66,393	75,392
Test	31,806	46,119	110,656	65,662

**TABLE 2 |:** Ranges of hyperparameters explored for different models (Engine Dynamometer and Chassis Dynamometer).

Dataset	Engine dynamometer		Chassis dynamometer	
Model	Engine-out *NO*_*x*_	Tailpipe *NO*_*x*_	Engine-out *NO*_*x*_	Tailpipe *NO*_*x*_
Learning Rate	[0.01,0.001,0.0001]	[0.01,0.001,0.0001]	[0.01,0.001,0.0001]	[0.01,0.001,0.0001]
Batch Size	[100, 500, 1,000, 95,417]	[100, 500, 1 000, 95,417]	[1,000, 5,000, 10,000, 3,31,967]	[1,000,5,000, 10,000, 3,31,967]
Input Layer Nodes	8	5	9	5
Hidden Layers	[2,3,4,5,6]	[2,3,4,5,6]	[2,3,4,5,6]	[2,3,4,5,6]
First Hidden Layer Nodes	[200,100,50,20]	[200,100,50,20]	[200,100,50,20]	[200,100,50,20]
Last Hidden Layer Nodes	[20,15,10,5]	[20,15,10,5]	[20,15,10,5]	[20,15,10,5]
Hidden Layer Activation Function	ReLU	ReLU	ReLU	ReLU
Output Layer Nodes	1	1	1	1
Output Layer Activation Function	ReLU	ReLU	ReLU	ReLU
Epochs	200	200	200	200

**TABLE 3 |:** Final optimal hyperparameters for engine-out and tailpipe *NO*_*x*_ models for dataset 1 and 2.

Dataset	Engine dynamometer		Chassis dynamometer	
Model	Engine-out *NO*_*x*_	Tailpipe *NO*_*x*_	Engine-out *NO*_*x*_	Tailpipe *NO*_*x*_
Input Layer Nodes	8	5	9	5
Hidden Layer Nodes	[200, 100, 50, 5]	[1,000, 500, 250, 100, 5]	[1,000, 500, 250, 100, 5]	[2,000, 1,000, 500, 250, 100, 5]
Hidden Layer Activation Function	ReLU	ReLU	ReLU	ReLU
Output Nodes	1	1	1	1
Output Layer Activation Function	ReLU	LeakyReLU	ReLU	LeakyReLU
Learning Rate	0.001	0.001	0.001	0.001
Learning Rate Decay	1/5 every 200 Epochs	1/5 every 200 Epochs	1/10 every 400 Epochs	1/10 every 400 Epochs
Drop Out	0	0.1	0.1	0.1
Batch Size	500	500	1,000	1,000
Epochs	600	600	1,000	1,000

**TABLE 4 |:** Evaluation metrics for train, validation and test set with 95% confidence intervals (all models).

Dataset	Engine dynamometer		Chassis dynamometer	
Model	Engine-out *NO*_*x*_	Tailpipe *NO*_*x*_	Engine-out *NO*_*x*_	Tailpipe *NO*_*x*_
Train MSE (g/s)	5.02E–06 ± 3.03E–07	7.79E–07 ± 2.14E–07	1.91E–05 ± 3.23E–07	4.21E–05 ± 2.07E–06
Val MSE (g/s)	7.35E–06 ± 2.47E–07	1.27E–06 ± 3.29E–07	4.30E–05 ± 8.60E–07	7.20E–05 ± 2.70E–06
Test MSE (g/s)	7.41E–06 ± 1.76E–07	1.43E–06 ± 3.37E–06	4.19E–05 ± 4.86E–07	7.07E–05 ± 9.22E–07
Train MAE (g/s)	1.22E–03 ± 2.41E–05	4.30E–04 ± 1.36E–04	2.48E–03 ± 2.61E–05	3.18E–03 ± 3.56E–05
Val MAE (g/s)	1.25E–03 ± 1.43E–05	4.44E–04 ± 1.32E–04	3.25E–03 ± 2.04E–05	3.94E–03 ± 7.02E–05
Test MAE (g/s)	1.34E–03 ± 1.77E–05	4.51E–04 ± 1.35E–04	3.27E–03 ± 2.20E–05	3.91E–03 ± 3.14E–05
Train *R*^*2*^	0.998 ± 0.001	0.996 ± 0.001	0.987 ± 0.001	0.956 ± 0.002
Val *R*^*2*^	0.997 ± 0.001	0.995 ± 0.001	0.971 ± 0.001	0.926 ± 0.003
Test *R*^*2*^	0.997 ± 0.001	0.994 ± 0.001	0.972 ± 0.001	0.927 ± 0.001
Train MAE (%)	0.512 ± 0.010	0.080 ± 0.025	0.516 ± 0.021	1.487 ± 0.040
Val MAE (%)	0.566 ± 0.006	0.084 ± 0.025	0.950 ± 0.020	2.137 ± 0.222
Test MAE (%)	0.566 ± 0.006	0.085 ± 0.025	0.883 ± 0.028	1.797 ± 0.020
Train Total *NO*_*x*_ Error (%)	0.086 ± 0.049	1.229 ± 1.250	0.116 ± 0.114	0.151 ± 0.231
Val Total *NO*_*x*_ Error (%)	0.100 ± 0.058	1.304 ± 1.375	0.155 ± 0.114	0.368 ± 0.350
Test Total *NO*_*x*_ Error (%)	0.084 ± 0.059	1.214 ± 1.313	0.167 ± 0.109	0.249 ± 0.195

## Data Availability

The raw data supporting the conclusions of this article will be made available by the authors, without undue reservation.
